# A minimal model of panimmunity maintenance by horizontal gene transfer in the ecological dynamics of bacteria and phages

**DOI:** 10.1073/pnas.2417628122

**Published:** 2025-08-01

**Authors:** Wenping Cui, Jemma M. Fendley, Sriram Srikant, Boris I. Shraiman

**Affiliations:** ^a^Kavli Institute for Theoretical Physics, University of California, Santa Barbara, CA 93106; ^b^Department of Physics, University of California, Santa Barbara, CA 93106; ^c^Department of Biology, Massachusetts Institute of Technology, Cambridge, MA 02139

**Keywords:** ecoevolutionary dynamics, horizontal gene transfer, panimmunity

## Abstract

Bacteria and phages have been in an ongoing arms race for billions of years. To resist phages bacteria have evolved numerous defense systems, which nevertheless are still overcome by counterdefense mechanisms of specific phages. These defense/counterdefense systems are a major element of microbial genetic diversity and have been demonstrated to propagate between strains by horizontal gene transfer (HGT). It has been proposed that the totality of defense systems found in microbial communities collectively form a distributed “pan-immune” system with individual elements moving between strains via ubiquitous HGT. Here, we formulate a Lotka–Volterra type model of a bacteria/phage community interacting via a combinatorial variety of defense/counterdefense systems and show that HGT enables stable maintenance of diverse defense/counterdefense genes in the microbial pan-genome even when individual microbial strains inevitably undergo extinction. This stability requires the HGT rate to be sufficiently high to ensure that some descendant of a “dying” strain survives, thanks to the immunity acquired through HGT from the community at large, thus establishing a new strain. This mechanism of persistence for the pan-immune gene pool is fundamentally similar to the “island migration” model of ecological diversity, with genes moving between genomes instead of species migrating between islands.

## Significance

Microbial pan-genomes contain remarkable diversity of defense and counterdefense genes that emerge from the perpetual arms race between bacteria and bacteriophages. While an individual genome cannot carry defenses against all phages, the panimmunity hypothesis posits that bacteria can acquire necessary defense genes from the environment by horizontal gene transfer (HGT). Here, we develop a minimal model of panimmunity, which computationally shows that HGT, at a sufficiently high rate, can enable stable maintenance of a diverse set of defense/counterdefense genes in the community pan-genome. The diversity persists even under the condition of continuous turnover of both bacterial and phage genomes, characterized by boom/bust cycles with frequent extinction of individual strains.

Bacterial viruses (phages) are the most abundant and diverse organisms on the planet. They exert substantial selection pressure on microbial communities by predation. For instance, it is estimated that marine viruses kill about 20% of all ocean microbes each day (1). Facing phage predation, bacteria have developed different defense systems and strategies to interrupt the phage replication process, including restriction-modification (RM), abortive infection, and CRISPR-Cas systems (2, 3). On the other side of this evolutionary conflict (4), phages have evolved counterdefense genes like the RM-inhibiting ocr (5, 6), protein inhibitors like dmd or tifA against abortive infection toxin–antitoxin systems (7, 8), and many different anti-CRISPR genes (9, 10).

Phages and bacteria have been a useful model to study evolution in the lab since the beginning of molecular biology. Although long-term coexistence can arise under conditions of genetic or phenotypic stratification (11, 12), or in spatially heterogeneous environments (13), coculturing experiments in the lab often result in either phages or bacteria fixing in the culture driving the other to extinction in the long term (14, 15). Further, mathematical models of such systems tend to show that an advantageous bacterium or phage would always dominate, with the ecosystem losing diversity in the long run (16, 17), unless given unrealistically high mutation rates (18, 19). However, we know that the evolutionary conflict has persisted in nature for billions of years, leading to the phylogenetic diversity we see today. Understanding the persistent coexistence of diverse bacteria and phages must involve stochastic ecological dynamics coupled with evolution, in addition to the genetic systems that the two sides use against the other (20).

Recent studies have found that many defense systems are colocalized on bacterial genomes in so-called defense islands (3, 21), and some are located on mobile genetic elements (22, 23). It has also been noted that the phylogeny of orthologous defense genes is discordant with the phylogeny of the core genomes of the strains that harbor them (24, 25), suggesting frequent horizontal transfer. The apparent mobility of defense systems is not surprising given that they are beneficial only in the presence of certain phages, while presenting a persistent cost, e.g. in metabolic resources of a cell. Given the diversity and abundance of defense systems, a given genome contains only a subset of existing defense genes (22, 23, 26). Evidence of frequent gain and loss of defense systems, along with the fact that in natural ecosystems the defense “arsenal” is distributed across strains, has motivated the panimmunity hypothesis, which suggests the view of the bacterial pan-genome as a collective “immune system,” with the diversity of defense systems maintained by transient selection and frequent horizontal gene transfer (HGT) (24, 27–29). Phages are also limited in the number of counterdefense genes they contain due to the constraint on their packaged genome size, and therefore are limited in the number of bacterial strains they can successfully infect (8). Thus, phages must evolve by gaining different combinations of counterdefense genes to continue to persist in a bacterial community that is shuffling a per-genome immune profile (4, 28, 29).

The panimmunity hypothesis, while not yet empirically established, extends the concept of diversity into multilevel space (30). Many of the existing models primarily focus on diversity at a single level, typically the number of surviving species (31–34). However, the panimmunity hypothesis emphasizes the importance of considering the diversity of genes and genomes (strains)—combinatorial sets of different genes—separately. Since the gene space is considerably smaller than that of genotypes, it becomes more achievable for diverse genes to persist over time, regardless of the rapid extinction and turnover of genomes.

Toxin–antitoxin (TA) systems are widely distributed across bacterial genomes, with diverse TA systems playing a role in phage defense (35). In TA systems, the toxin activity, that is normally inhibited by the antitoxin, is triggered by phage infection, leading to a collapse of phage replication. Given the mechanistic diversity and abundance of these systems in bacterial genomes, phages must evolve to evade a wide variety of TA systems. One evolutionary strategy phages have used is to maintain antitoxins to inhibit TA systems and allow infection of bacteria (7, 8). Phages have even been shown to acquire the antitoxin from the TA locus by HGT to evolve the ability to infect the bacteria (36). This is possible as bacterial immune systems do not provide absolute protection against phage infection. TA systems can therefore play a key role in phage–bacteria coevolutionary dynamics providing an immune barrier to viral replication but also providing the basis of viral resistance by HGT.

In this article, we develop a stochastic Lotka–Volterra (LV) model that incorporates HGT between genotypes and explores the feasibility of panimmunity. Our minimal model can exhibit typical LV behaviors: demographic-noise-driven extinction (37) and persistent (oscillatory) coexistence, realized for different HGT rates. Surprisingly, between these two phases, we identify another regime where the panimmunity hypothesis operates, and genes can persist along with the “boom-bust” dynamics of individual strains or particular genotypes. Inspired by recent theoretical progress in islands-migration models (38), we derive criteria for the gene and genotype persistence regimes. Our results suggest that a relatively small HGT rate, inversely proportional to the total population size, is sufficient to maintain the coexistence of diverse genes, independent of the combinatorial diversity of genotypes, explaining how the numerous defense and counterdefense systems can persist despite strong selections in nature (39, 40).

## Model

We shall focus on bacteria–phage interactions assuming for simplicity that susceptibility to specific phage infection is the only heritable phenotype that affects relative fitness of bacterial species, and similarly, the ability to infect a specific bacterium is the only relevant distinction between phages. Thus, the relevant space of genotypes of bacteria and phages is defined respectively by the defense and counterdefense genes.

To illustrate the effect of HGT, it will suffice to frame our model in the context of the toxin/antitoxin (TA) paradigm of phage defense (35). The key elements of TA systems can be abstracted in terms of a toxin gene, *A*, and its specific antitoxin, *a*. A bacterium carrying A must also carry a and, we shall assume, can only be infected by a phage carrying the antitoxin a. We shall posit the existence of a large number of distinct, specific toxin/antitoxin pairs denoting them by L different uppercase/lowercase letters with L≫1. We emphasize that on this level of abstraction, the toxin/antitoxin paradigm captures the general aspects of a broad variety of actual defense/counterdefense systems, without delving into the biochemical and genetic complexity of their mechanisms. This will suffice for our goal of providing a mathematical underpinning for the “panimmunity” hypothesis.

**Fig. 1.**
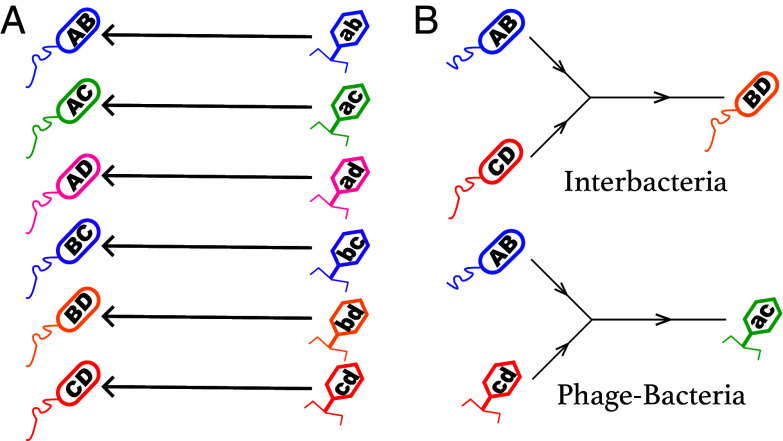
Model bacteria and phage genotypes and HGT processes. (*A*) Interactions between bacteria and phages for the case of L=4 different TA systems. The capital letter denotes a specific toxin in the bacterium; the lowercase letter denotes a corresponding antitoxin gene in the phage. Bacterium AB will carry the toxins A and B, in addition to the antitoxins a and b. (*B*) Scheme for the new clones formed by interbacteria and phage–bacteria HGT processes.

Each bacterium and phage can carry multiple defense and counterdefense genes respectively, introducing combinatorial complexity into ecological interactions. We start with each bacterial and phage strain carrying just two distinct TA genes. Correspondingly, there are K=L(L−1)/2 possible genotype combinations in total. Fig. 1*A* shows an example of L=4, where there are K=6 possible bacteria–phage pairs.

Let $B_{\mathrm{ij}}$ and $V_{\mathrm{ij}}$ denote the population sizes of bacterial and phage strains (number of individuals in the system), respectively, with index ij specifying the genotype via the labels i,j of its TA genes. By construction, $B_{\mathrm{ij}}$ and $V_{\mathrm{ij}}$ are symmetric matrices, and i≠j. The ecoevolutionary dynamics of bacteria and phage populations may be described by the generalized LV equations:

[1a] $$\begin{array}{cc} \frac{dB_{\mathrm{ij}}}{\mathrm{dt}} & =sB_{\mathrm{ij}}-\frac{\phi }{N_{B}}B_{\mathrm{ij}}V_{\mathrm{ij}}+\frac{r_{B}}{2N_{B}}\sum_{k,l}B_{\mathrm{ik}}B_{\mathrm{lj}}, \end{array}$$

[1b] $$\begin{array}{cc} \frac{dV_{\mathrm{ij}}}{\mathrm{dt}} & =\frac{\beta \phi }{N_{B}}B_{\mathrm{ij}}V_{\mathrm{ij}}-\omega V_{\mathrm{ij}}+\frac{r_{V}}{4N_{B}}\sum_{k,l}\left(V_{\mathrm{ik}}B_{\mathrm{lj}}+V_{\mathrm{lj}}B_{\mathrm{ik}}\right), \end{array}$$

where s is the per capita bacterial division rate, β is the burst size (dimensionless), and ω is the phage’s per capita death rate. $\phi /N_{B}$ is the successful per capita infection and lysis rate so that the infection term does not scale with the number of individuals in the system, and $N_{B}$ is the total number of all bacterial individuals, which remains undetermined. For simplicity, we neglect the potential dependence of these on TA genotype ij, effectively assuming that all TA systems have the same intrinsic fitness cost, so that each phage/bacterium pair follows similar dynamics.

The final terms in Eq. **1** represent the HGT process. As a minimal model—a simplified view of bacterial conjugation—we assume that a bacterium can replace one of its genes with a gene from another bacterium. Fig. 1*B*, *Top* shows the bacterial recipient *CD* randomly acquires TA gene *B* from some donor *AB* and transforms its genotype to *BD*. Antitoxin genes can be passed on from one phage to another, via instances of coinfection of the same bacterium. Alternatively, they can be passed directly from a resistant bacterium to a phage genome (8). This is possible since bacterial immune systems are not perfect barriers to viral infection (36). Fig. 1*B*, *Bottom* shows the latter. We are interested in the total number of transfers into a given strain per unit time, which is also measurable by statistical inference from experimental data (41), rather than the rate per unit volume, as in mass action. Therefore, we define bacteria and phages to have per capita HGT rates $r_{B}$ and $r_{V}$, with the respective terms shown in Eq. **1**.

To investigate extinction driven by demographic noise, we generalize Eq. **1** into a Poisson process framework. Specifically, bacteria and phages now follow stochastic growth with fitness defined by $s-\phi V_{\mathrm{ij}}/N_{B}$ and $\beta \phi B_{\mathrm{ij}}/N_{B}-\omega$, respectively. As the number of HGT events is proportional to the population size, it is convenient to reparameterize the bacterial and viral “fitness” terms, respectively, as $s(1-V_{\mathrm{ij}}/n_{V}^{*})$ and $\omega (B_{\mathrm{ij}}/n_{B}^{*}-1)$, where $n_{V}^{*}=sN_{B}/\phi$ and $n_{B}^{*}=\omega N_{B}/\left(\beta \phi \right)$ are the phage and bacteria characteristic population sizes for each strain. For scaling purposes, we introduce the ratios $\rho_{n}=n_{V}^{*}/n_{B}^{*}$, $\rho_{s}=\omega /s$, and $\rho_{r}=r_{V}/r_{B}$ and rewrite $n_{B}^{*}=n_{G}^{*}$, and $r_{B}=r$. The two-species LV dynamics can always be rescaled to the antisymmetric form (38, 42). To further simplify our analysis, we initially investigate the perfectly antisymmetric form by setting $\rho_{s}=\rho_{n}=\rho_{r}=1$. The summary of notations can be found in *SI Appendix*, section 1. In *SI Appendix*, section 7 we show that our primary findings still hold in the more general parameter setting, and the case $\rho_{r}\neq 1$ is considered later in Fig. 5.

However, the total population sizes $N_{B}=\frac{1}{2}\sum_{i\neq j}B_{\mathrm{ij}}$, and $N_{V}=\frac{1}{2}\sum_{i\neq j}V_{\mathrm{ij}}$ are still undetermined and depend on complex relationships involving all system parameters. To avoid solving sophisticated self-consistency relations for $N_{B}$ and $N_{V}$, we impose a hard constraint on the total population sizes. First, we initialize s, ω, $N_{B}$, and $N_{V}$ and then take $n_{B}^{*}=N_{B}/K$ and $n_{V}^{*}=N_{V}/K$. The population size constraint is achieved by introducing regulators which tune *s* and ω. Notably, we find that the collective phenomena associated with gene transfer are insensitive to s and ω as they either cancel out or introduce order-1 corrections in most cases. The full description of our simulation can be found in *SI Appendix*, section 2.

Biologically, the fixed total population size serves as a universal carrying capacity across strains. Our study focuses on strains competing within the same ecological niche, distinguished solely by their phage defense systems. Consequently, we adopt LV dynamics with fixed total population sizes, neglecting strain-specific carrying capacity terms in conventional consumer–resource models. Without phages, our model becomes the Wright–Fisher model (43); bacterial strains undergo random drift because of the finite population size, one strain dominates, and the ecosystem completely loses genetic diversity.

## Results

### Different Regimes in the Dynamics of Genes and Genotypes.

**Fig. 2.**
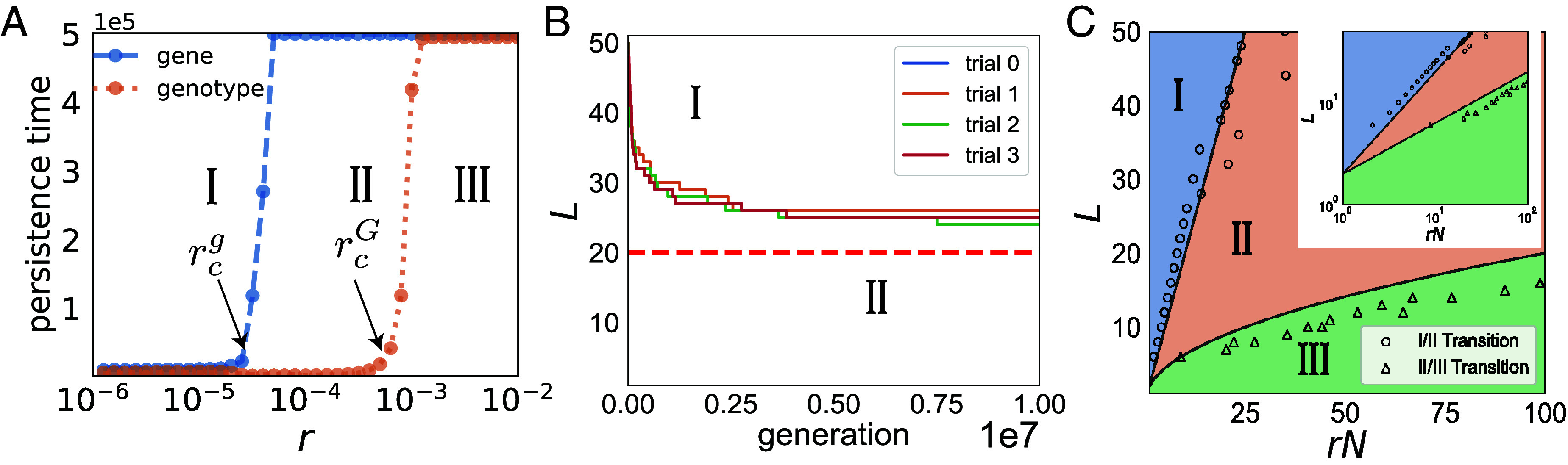
Different regimes of HGT-driven bacteria/phage dynamics. (*A*) Persistence time for genes and genotypes under different HGT rate r (and fixed $N=10^{6}$ and initial L=40). Persistence time is defined by the simulation time (averaged over 5 trials) until the first gene or genotype is lost (with the upper limit at generation $T=5\times 10^{5}$ imposed by the maximum simulation length). (*B*) Genetic diversity decreases when its initial value exceeds $L_{\text{max}}$ (for $N=10^{6}$ and $r=2\times 10^{-5}$). The solid lines are from 4 trials of simulations. The red dashed line is $L_{\text{max}}\propto rN$ given by our theory. (*C*) Parameter regimes corresponding to three qualitatively different behaviors: I: unstable coexistence; II: persistence of genes with continuous turnover of genotypes; and III: stable coexistence. Each circle and triangle marker represents one-trial simulations to search for the transition boundary between I/II and II/III at given parameters. The *Inset* shows the same data but on a log–log scale. The black solid lines separating different regimes are our theoretical predictions given by Eq. **7**: $L_{\text{max}}\propto rN$ (*Upper*) and $K_{\text{max}}\propto rN$ (*Lower*), respectively. The prefactors are estimated empirically. Our default simulation parameters are $N=10^{6}$, L=40, K=780, and $s=5\times 10^{-3}$ unless specified otherwise.

We would like to understand how HGT processes affect the distributions of bacteria and phage genotypes and the frequencies of specific TA genes in the pan-genome. Fig. 2*A* shows how long the system keeps its full gene and genotype diversity when all genotypes start from the mean population size N/K, with $N=N_{B}=N_{V}$. For simplicity, we set $r=r_{B}=r_{V}$, as described in the previous section. Numerical simulations reveal three distinct regimes as a function of the HGT rate r, and thus two critical HGT rates $r_{c}^{g}$ and $r_{c}^{G}$ are defined with $r_{c}^{g}<r_{c}^{G}$.

Regime I is the unstable-coexistence regime which occurs for low HGT rate $r<r_{c}^{g}$. In this regime any given strain (i.e. a particular ij genotype) persists for a short time before going extinct and is unlikely to reappear via HGT. Loss of genotypes leads to a loss of genes (Fig. 2*A*), reducing genetic diversity. The bacteria/phage system keeps losing genetic diversity until the “birth rate” of genotypes via HGT is sufficient to compensate the loss of genotypes under selection. The genetic diversity will then be sustained, but with a smaller number of genes (i.e. TA systems), $L_{\text{max}}$ (Fig. 2*B*), and a larger average population size of different genotypes. Thus, the unstable-coexistence regime appears transiently while $L>L_{\text{max}}$, as shown in Fig. 2*B*, with the dependence of $L_{\text{max}}$ on rN defining the boundary of the unstable coexistence regime in the phase diagram Fig. 2*C*.

Regime II is the regime of genotype turnover and gene persistence, which occurs in the intermediate range of HGT rate $r_{c}^{g}<r<r_{c}^{G}$. In this regime, bacteria–phage pairs undergo boom-bust behavior: A newly established bacterial genotype, formed by HGT, grows rapidly until infected by a phage which subsequently leads to a “crash” and then goes extinct.

**Fig. 3.**
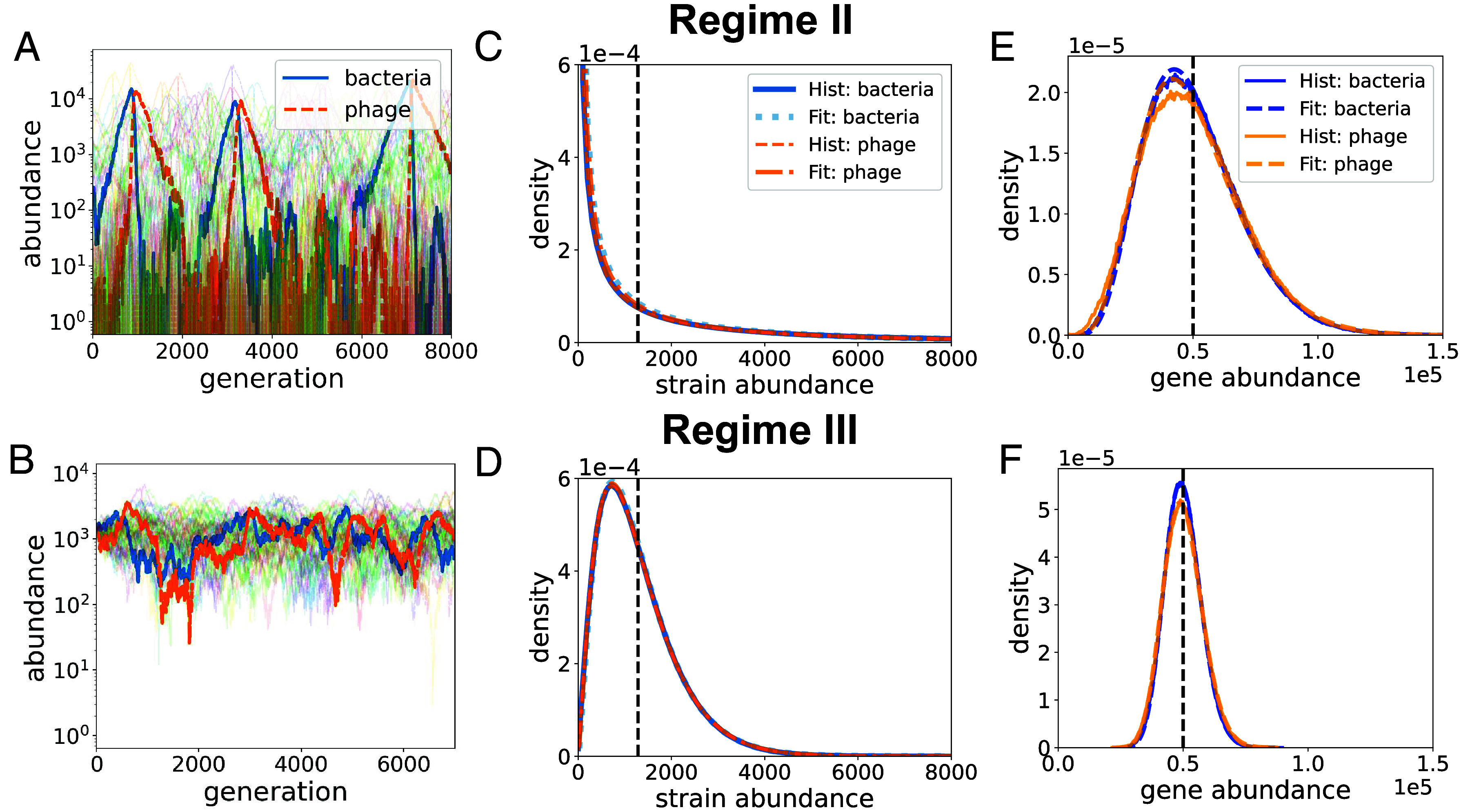
Phage/bacteria population dynamics and the distributions of genes and genotypes in Regimes II and III. (*A* and *B*) Time course of multiple phage (orange dashed lines) and bacteria (blue solid lines) populations. One phage/bacteria-pair is emphasized to illustrate distinct dynamics in Regime II ($r=5\times 10^{-5}$) and Regime III ($r=10^{-3}$) in (*A* and *B*) respectively. Corresponding genotype (*C* and *D*) and gene (*E* and *F*) abundance distributions. Dotted and dash-dotted lines are the fitted Gamma distributions; the lines overlap so extensively that they are difficult to distinguish from one another. The vertical lines are the mean clone size. The vertical lines are the mean clone size for *B* and *E* and the mean gene population size for *E* and *F*.

Critically, however, in this regime the HGT rate is high enough for one or more descendants of the clonal population to acquire immunity against the phage, via a horizontally transferred TA gene. A successful transfer event establishes a new immune genotype, which will subsequently undergo a boom-bust cycle of its own. Fig. 3*A* shows that the genotypes have a short lifetime, but HGT enables TA genes to persist in the pan-genome of the community, effectively “surfing” from one boom-bust wave to another. This regime provides a model for the panimmunity hypothesis. Only a fraction of all possible genotypes exist at any instant of time; however, the system can still maintain genetic diversity by distributing genes across the community.

Regime III is the regime of genotype persistence realized at a sufficiently high HGT rate $r>r_{c}^{G}$. Fig. 3*B* shows the bacteria–phage pair undergoing stochastic oscillations around a well-defined mean population size without going extinct. In this regime frequent HGT spreads bacterial and phage genotypes over all available genotypes, making immune escape impossible, while also eliminating large boom-bust events. This is the endemic infection regime, which is also the regime of stable bacteria–phage coexistence.

Our simulations further demonstrate that the critical HGT rates $r_{c}^{g}$ and $r_{c}^{G}$ most sensitively depend on the total population size N and the genetic diversity L. Consequently, we can alternatively express the critical curves for a given N and r in terms of $L_{\text{max}}$, representing the maximum genetic diversity and analogous to the carrying capacity in ecology. If the initial value of L is larger than $L_{\text{max}}$, the system starts at Regime I, which is unstable, and subsequently slides toward the edge of Regime II, as illustrated in Fig. 2*B*. Likewise, we also have the maximum genotypic diversity $K_{\text{max}}$, conditioned upon the coexistence of all possible genotypes, lying at the boundary between Regime II and Regime III. Fig. 2*C* provides the full phase diagram (we transform $K_{\text{max}}$ back to its corresponding $L_{\text{max}}$ for comparison in the same diagram), and we will give our theoretical analysis of the critical curves in the following sections.

### Phage/Host “Ensemble”.

What is the critical value of r for the transition between regimes, given model parameters? To identify suitable observable quantities to describe the highly nontrivial population dynamics of many phage–bacteria pairs in Fig. 3 *A* and *D*, we shall follow Goel. et al. as our system in Eq. **1** falls into a broad class of antisymmetric LV models investigated before (38, 42). It is useful to define the Lyapunov function (42) for a single bacteria–phage pair as

[2] $$\begin{array}{c} E\left(B,V\right)=\left(B-n_{G}^{*}\log\frac{B}{n_{G}^{*}}\right)+\left(V-n_{G}^{*}\log\frac{V}{n_{G}^{*}}\right), \end{array}$$

which characterizes the distance between the current state and the steady state. In the deterministic limit N→∞, E is determined by the initial condition and does not change with time, corresponding to a bacteria/pathogen population undergoing a neutrally stable periodic oscillation.

In the spirit of statistical mechanics, one may interpret E as the energy of a “particle,” and describe the state of the stochastic LV system with a large number of interacting strains by a canonical ensemble (42, 44). In our case HGT processes play a role similar to particle collisions for energy exchange. Then the probability density function (PDF) of microstates follows Boltzmann statistics which give the Gamma distribution (see *SI Appendix*, section 3 for details):

[3] $$\begin{array}{c} n_{G}∼\Gamma \left(n_{G}^{*}=\frac{N}{K},\Theta \right)=\frac{n_{G}^{\frac{N}{K\Theta }-1}e^{-n_{G}/\Theta }}{\Theta^{\frac{N}{K\Theta }}\Gamma \left(\frac{N}{K\Theta }\right)}. \end{array}$$

For simplicity, we use $n_{G}$ to represent either bacteria or phage abundances since their distributions have the same general form. The mean $n_{G}^{*}$ is given by N/K, and Θ is an unknown effective temperature for genotypes, characterizing the fluctuations of genotype abundances.

The gene abundance $n_{g}$ is the sum of population sizes of all existing genotypes containing a specific gene g. We assume that the Gamma distribution ansatz can also be applied to the gene abundance: $n_{g}∼\Gamma \left(2N/L,\theta \right)$, where 2N/L is the mean gene abundance (the factor 2 arising from two-gene genotypes), and θ is another unknown effective temperature for genes.

Fig. 3 shows that Gamma distributions provide an excellent fit to the genotype and gene abundance distributions across Regime II and III, even though their population dynamics behave quite differently.

### “Effective Temperatures” for Genes and Genotypes.

We next address the relationship between the effective temperatures, θ and Θ, that describe phage/bacteria statistics within the canonical ensemble framework, with the parameters controlling phage/bacteria dynamics.

The HGT process can be approximated in the mean-field sense by treating the recombination terms as constant rN/K source terms in both Eq. **1**. This is because the sampling probability of a specific genotype from HGT is proportional to the product of marginal probabilities of genes it carries (45); at the genotype scale, the fluctuations of gene abundances can be averaged out with the law of large numbers (also see simulations in *SI Appendix*, Fig. S12), suggesting that the new clones can be uniformly sampled over the whole genotype space. Within this approximation, the effect of HGT on ecological dynamics is similar to the effect of “island migration,” extensively studied in the context of LV models of ecological dynamics (38).

Using Itô’s lemma (46) and the mean-field approximations, we can write down the Lyapunov function dynamics for Eq. **1** with additional terms for demographic noise:

[4] $$\begin{array}{c} \begin{array}{cc} dE= & \left(\frac{n_{G}^{*}}{2B}+\frac{n_{G}^{*}}{2V}-r\frac{N(n_{G}^{*}-B)}{\mathrm{KB}}-r\frac{N(n_{G}^{*}-V)}{\mathrm{KV}}\right)dt\\ & +\sqrt{\frac{{\left(B-n_{G}^{*}\right)}^{2}}{B}+\frac{{\left(V-n_{G}^{*}\right)}^{2}}{V}}d\eta . \end{array} \end{array}$$

From the above equation, we see that demographic noise contributes a positive drift and drives E to grow exponentially (*SI Appendix*, section 4). The injection of new clones due to HGT processes contributes a balancing force to cancel the effect of demographic noise and stabilizes the system.

Without HGT, the demographic noise drives the system away from the steady state. The oscillation amplitude grows until either the bacterium or the phage dies out and the coexistence becomes unstable after a typical persistence time proportional to the average clone size N/K (*SI Appendix*, section 4 and Fig. S2). Once any phage goes extinct first, its corresponding bacterium becomes advantageous and drives the catastrophic extinction of other bacteria because of competitive exclusion.

In Regime III where r is sufficiently large, the system is stable, and the average of E does not change in thermal equilibrium. We then obtain $\Theta =\frac{1}{2r}$ by solving the self-consistency relation that the drift part averaged over the canonical ensemble is zero. With similar calculations for the mean-field gene dynamics derived from quasi-linkage equilibrium (45), we obtain $\theta =\frac{2}{r}$, larger than Θ. This is because the sampling space of the interbacteria HGT tends to concentrate on the genotypes containing abundant genes. The biased sampling can be written as a quadratic term in the mean field approximation, resulting in stronger fluctuations at the gene level than the genotype level (*SI Appendix*, section 5 for more details).

However, our previous analysis does not work for Regime II as either the phage or the bacterium can go extinct, and the Lyapunov function E is no longer well defined. Fig. 3 *A* and *C* shows that most strains have small population sizes, represented by a large pile-up near zero, while a few booming strains dominate in the system, represented by an exponential tail on the right. We can focus on the booming strains, and the exponential tail in the Gamma distribution suggests they have a typical population size (peak size) ∼Θ (38).

We next estimate the typical peak size of the booming strains by connecting the “kill-the-winner” mechanism (18, 47, 48) with the establishment probability in population genetics (49, 50) as follows: The booming bacterial strain has an approximately constant fitness s as long as its corresponding phage population is small. Hence, the bacterial strain (after establishing itself with the population size $\frac{1}{2s}$) follows deterministic exponential growth $e^{\mathrm{st}}$ until the phage with antitoxin genes that allow infection of this bacterial strain emerges due to HGT and is itself established (49) (*SI Appendix*, Fig. S3). Since the boom-bust cycles show that, most of the time, the phage does not impede the exponential growth of the susceptible bacterial strain until its establishment, we can use branching processes to evaluate the phage’s establishment probability conditioned on the susceptible bacterial strain’s instantaneous abundance (51). Then considering the constant rebirth rate rN/K for one specific phage strain along with its probability of establishment, we can estimate the waiting time for the first phage to get established (49). Upon establishment, given the large abundance of the bacterial strain at this time, the established phage proliferates rapidly, leading to the immediate decline of the susceptible bacterial strain population. Consequently, the bacterial strain population size at the time of the phage’s establishment can be used to determine its typical peak size. Our analysis (see *SI Appendix*, section 6 and Fig. S4 for details) shows that the peak size follows an exponential distribution with exponential rate −2r, yielding $\Theta \approx \frac{1}{2r}$. In this regime, as r is small, different booming strains are weakly correlated and the gene abundance distribution is approximated by the sum of L−1 independent Gamma distribution for genotypes, which is still a Gamma distribution with the same exponential tail, yielding $\theta =\Theta =\frac{1}{2r}$.

**Fig. 4.**
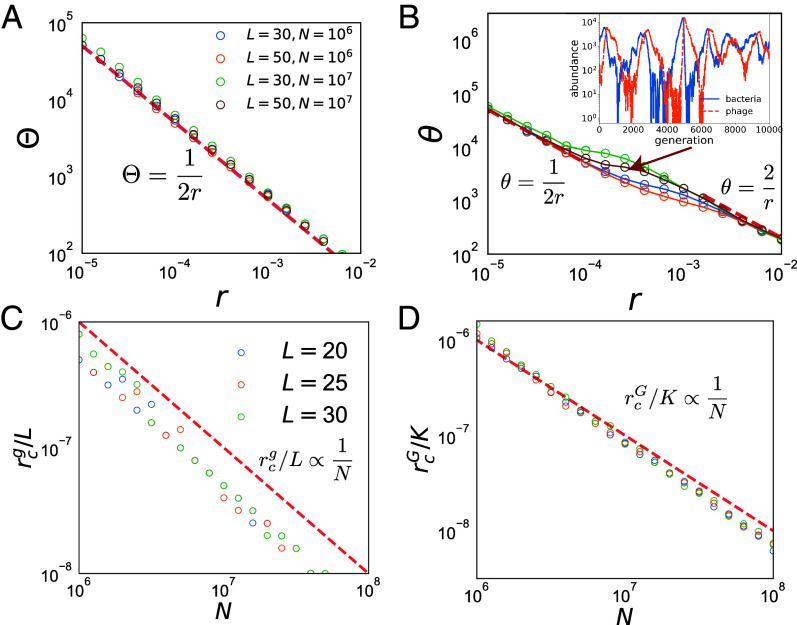
“Effective temperatures” and critical HGT rates. (*A* and *B*) The scaling of Θ and θ in Gamma distributions of genotype and gene abundances at different HGT rates r. Red dashed lines are theoretical predictions. *Inset* in (*B*) shows an example of a phage–bacteria pair exhibiting a mixture of boom-bust and stochastic-oscillation dynamics at the crossover between Regimes II and III. (*C* and *D*) Critical (minimal) HGT rates for gene and genotype coexistence. The red dashed lines are our predictions. All data points are simulations with gene (*C*) or genotype (*D*) persistence time over $2\times 10^{5}$ generations.

In summary, our analysis of the dynamical behavior suggests the following scaling:

[5] $$\begin{array}{c} \left\{\begin{array}{cc} \Theta =\theta =\frac{1}{2r}\; & \text{for boom-bust cycles}\\ \Theta =\frac{1}{2r},\;\theta =\frac{2}{r}\; & \text{for stochastic oscillations} \end{array}\right. \end{array}$$

which match well with numerics in Fig. 4 *A* and *B*. Fig. 4*B* also shows the system can exhibit mixed dynamics when θ changes smoothly from $\frac{1}{2r}$ to $\frac{2}{r}$ in the transition from Regime II to III, consistent with our theory.

### Critical Values of r.

The shapes of the PDF for genes and for genotypes provides a natural criteria designating different regimes: The power exponent in Eq. **3** must be positive in order to avoid the PDF diverging at $n_{G}=0$ or $n_{g}=0$, which requires that

[6a] $$\begin{array}{cc} \frac{N}{K\Theta }-1 & >0, \end{array}$$

[6b] $$\begin{array}{cc} \frac{2N}{L\theta }-1 & >0. \end{array}$$

Otherwise, the mode of the PDF is at zero, corresponding to an extinction of the genotype or gene. Hence, the equalities $\frac{N}{K\Theta }=1$ and $\frac{2N}{L\theta }=1$ demarcate transitions between I/II and II/III respectively. Given the scaling of Θ and θ in Eq. **5**, the minimal r to maintain gene and genotype diversity are

[7a] $$\begin{array}{cc} r_{c}^{g} & \propto L/N, \end{array}$$

[7b] $$\begin{array}{cc} r_{c}^{G} & \propto K/N. \end{array}$$

While this scaling is robust, the argument does not determine the prefactor (which is order 1); gene and genotype abundances fluctuate strongly near respective transitions, invalidating the mean-field theory assumptions that we have made in treating HGT (*SI Appendix*, Fig. S6). Overall, as shown in Fig. 4 *C* and *D*, Eq. **7** agree well with the results of numerical simulations. Alternatively, we can define transitions in terms of L as a function of the total population size N and the HGT rate r. Eq. **7** imply that $L_{\text{max}}$ and $K_{\text{max}}$ at the transitions are proportional to rN -a result confirmed by numerical simulations in Fig. 2*C*.

**Fig. 5.**
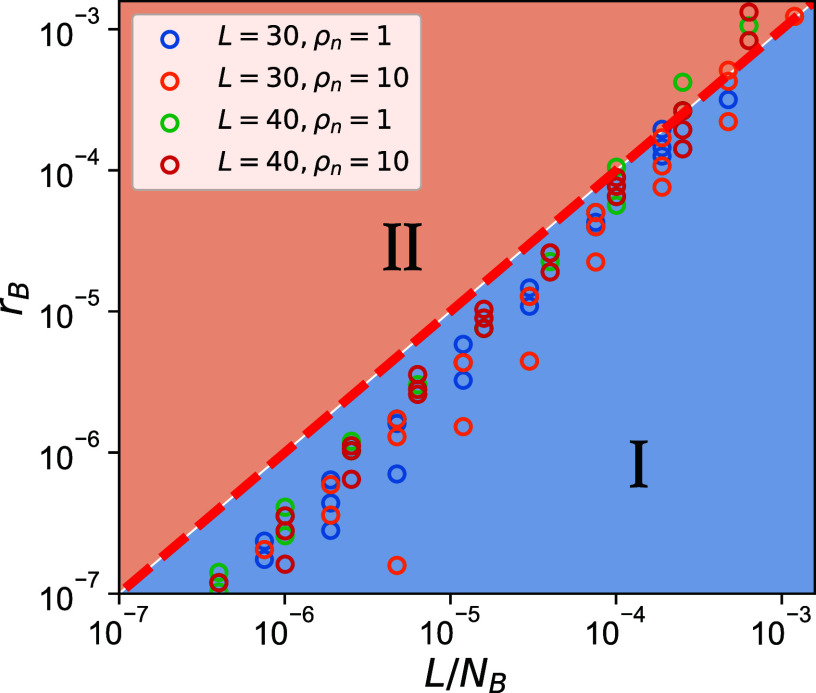
“Phase diagram” for unequal interbacteria and bacteria–phage HGT rates. The scatter points indicate the numerically determined transition points between Regions I and II. We varied $N_{B}$ from $10^{5}$ to $10^{8}$ with $\rho_{n}=N_{V}/N_{B}=1,10$ and $r_{V}=100/N_{B},200/N_{B},400/N_{B}$, ensuring the system stays in Regime II. The red dashed horizontal line: y=x is our theoretical estimate for the I/II boundary given by Eq. **8**.

In the preceding analysis, we set the interbacteria and bacteria–phage recombination rates to be equal, $r_{B}=r_{V}$, and derived the critical phage–bacteria HGT rate from the perspective of phage-induced growth inhibition. We now consider the case $r_{B}\neq r_{V}$, highlighting the importance of interbacterial HGT as a survival mechanism for “escaping” predation. In the limit of high $r_{V}$ when the system resides in Regime III, only negligible interbacteria HGT is required to maintain stable oscillatory behavior. To locate the boundary between Regimes I and II, we estimate the minimum interbacteria HGT rate required for bacterial populations to survive phage predation by acquiring a suitable defense gene. A bacterial clone with population size B(t) acquires genes at a rate $r_{B}B\left(t\right)$ and so that the expected number of established resistant offspring lineages generated during the “bloom” event is $sr_{B}\int dtB\left(t\right)$ which, to avoid extinction, should be >1. We denote the growth phase of the “booming” bacterial clone as $\tau_{\text{boom}}$ (the period from a bacterial strain’s establishment to its peak abundance), and we estimate the time integral using the average size of the booming clone, $N_{B}/n_{\text{boom}}$, where $n_{\text{boom}}$ is the average number of the booming bacterial strains in the system. This leads to the inequality: $N_{B}r_{B}s\tau_{\text{boom}}>n_{\text{boom}}$. In order to remain in Regime II where all genes are present, $n_{\text{boom}}>L$. We estimate $\tau_{\text{boom}}$ as the time required for a bacterial strain to grow from the established population size 1/s to the typical booming size ($N_{B}/L$) with the growth rate s. This estimation gives $s\tau_{\text{boom}}\approx \;\log\frac{sN_{B}}{L}$, which is of order 1 correction, leading to the simple relation (verified in Fig. 5):

[8] $$\begin{array}{c} r_{B}>L/N_{B}. \end{array}$$

It demonstrates that when the phage infection rate is sufficient yet not unreasonably high to suppress dominant bacterial growth, the “escape” rate for bacteria to maintain genetic diversity is necessary and comparable to the value given by Eq. **7a**. Notably, as it is derived at the critical point of defense gene extinction, this relation is dependent on $r_{B}$ and system dynamics are governed primarily by the bacterial “escape” rate rather than the phage “predation” rate at the boundary.

### Generalization.

The transition criteria defined above are quite general. As an example, we consider the transition to the gene maintenance regime for the case with “tripleton” genotypes, i.e. each genotype carrying three genes so that $K\propto L^{3}$ in contrast to $K\propto L^{2}$ for the “doubleton” case analyzed above. Fig. 4 and *SI Appendix*, Fig. S13 show that the HGT rate required to maintain genetic diversity only grows linearly with the number of genes, in contrast to the faster polynomial (in L) growth of the genotype space. We can understand this from our criterion for gene persistence, which gives rN as the estimate for the number of booming bacterial strains (each with a typical population size of order 1/r). This number must exceed L so that there are enough booming strains to carry all genes to escape from selective sweep, regardless of the “doubleton” or “tripleton” structure of the genotype.

As there are multiple ways for phages and bacteria to acquire genes (8), we also consider the effect of interphage HGT as the dominant mechanism for generating “new” phages (*SI Appendix*, section 7). The mean-field theory description of HGT as a constant source of genotypes still applies, and *SI Appendix*, Fig. S6 shows that Eq. **7** remain valid. Interphage transfer creates necessary escape genotypes by coinfection of a host by multiple phages each carrying one of the complementary antitoxins needed. The “biased sampling” effect of the bacterium-phage HGT does not apply, and we would expect that the infection rate required to maintain the gene pool in a high-dimensional genotype space must be substantially higher compared to the case of phage–bacteria transfer. This situation (which is unlikely when rN>L), is of course mitigated by any low rate of bacterium-phage HGT or new antitoxin emergence via de novo mutation (not considered in the present analysis).

Our previous analysis focused on antisymmetric and identical phage–bacteria pairs. However, in natural ecosystems, the phage/bacteria population size ratio $\rho_{n}∼10$, and the burst size $\beta =\rho_{n}\rho_{s}∼10-100$. Our approximate analysis (*SI Appendix*, section 8) reveals that increasing the burst size, the phage population size, and the phage–bacteria HGT rate generally strengthens the “kill-the-winner” mechanism and reduces the critical HGT rates required, which is validated through numerical simulations in *SI Appendix*, Figs. S8 and S9. We also demonstrate that our results are robust to the extension to nonidentical phage–bacteria pairs with parameters sampled from log-normal distributions (*SI Appendix*, Fig. S10).

Recognizing the biological limitations of the one-phage-one-bacteria assumption, we extended our analysis to a random sparse interaction matrix, in which phages can infect a random selection of bacteria (details in *SI Appendix*, section 9). Our results are robust to sparse interaction matrices, as shown in *SI Appendix*, Fig. S11. We anticipate that our results can be applied to marine microecosystems, where coisolation of phages and bacteria from the open ocean has revealed sparse infection profiles, with few broadly infective phage or resistant bacteria (27). As random sparse interaction matrices decouple genes from genotypes, we could also consider a scenario where specific antitoxin genes can inhibit multiple toxin genes. In our current model set-up, toxins inhibited by multiple antitoxins would have significantly lower fitness, and selection would drive them to extinction. In order to investigate the stability of these more complex interactions, we should also introduce distribution of fitness associated with toxins/antitoxins and allow variable gene number. This analysis is beyond the scope of this current paper and is an avenue for future exploration.

## Discussion

In this article, we have formulated and explored a simple model of phage–bacteria competition driven by bacterial defense and phage counterdefense systems, focusing on the role of HGT in maintaining gene diversity. Our model aims to explain the stability of diverse immune repertoires in bacterial populations as they coevolve with bacteriophages. Although our model is easiest to directly relate to toxin–antitoxin systems, genome cataloging has identified that Restriction-Modification (RM) and CRISPR-Cas systems are among the most abundant classes of defense systems in genomes across bacterial phylogeny (52, 53). Due to the diversity of proteins and domains that form TA systems, cataloging efforts do not properly account for the proportion of all the TA systems in phage defense, but an increasing number of TA system types continue to be implicated (35, 54). Like the presence of antitoxins on phage genomes to overcome immune barriers, modification enzymes homologous to those in RM systems (55) and CRISPR spacer-like elements (56) have been identified on phage genomes to overcome RM and CRISPR-Cas immunity. Further, it is expected that the source of these genes is from HGT-mediated acquisition from the RM and CRISPR systems of the bacterial host as part of the arms race. Thus the most abundant systems involved in immunity contribute to the arms-race evolution of phage–bacteria both in the distribution of the pan-immune system over the bacterial population and in the presence of counterdefense genes across phage genomes, consistent with our model of maintenance of genetic diversity through phage–bacteria gene transfer.

As described, our model is limited to understanding the “short-term” evolutionary process in phage–bacterial systems. Our analysis focused on the feasibility of maintaining diversity in the presence of frequent extinction (of individual strains and their genotypes) solely by the virtue of HGT without the effect of mutation. The latter is of course well known to play an essential role in bacterium-pathogen coevolution, e.g., mutations in bacterial and phage proteins abrogate or restore their specific interaction (57–59). The evolution of TA systems is also thought to proceed by mutations, gene-swapping, and possible de novo gene evolution, leading to rapid diversification and difficulty accounting for the number of systems associated with phage defense (35, 52, 54). Short, orphan genes in phage are associated with anti-immune function, and likely represent subsequent adaptation to overcoming bacterial defense (7, 8, 54, 60).

The importance of HGT processes in ecological dynamics and evolution of microbial communities is now broadly recognized (61). HGT appears to be ubiquitous in nominally asexual microbes with an estimated rate comparable to the rate of mutation (62). Frequent HGT effectively unlinks genes from genomes, enabling selection to act on genes in the community metagenome, much like it does in linkage quasi-equilibrium in sexually reproducing populations (45). In this sense, the pool of TA genes forming the panimmunity resource of the microbial community is maintained by selection through the boom/bust cycles of our Regime II.

Although little is presently known of the rates of recombination and genome turnover in ecological settings, lab experiments of evolving phages have given us a window into the possibilities. In the lab, when maintaining a phage population size of $10^{6}$ to $10^{8}$, it is possible to select evolved clones with recombination-based genome variation in 6 to 10 infection-generations (8). Such experiments have shown that phage genomes can amplify genes already present in their genome (8), acquire genetic material from coinfecting phage (63), and from the infected bacterial cell’s genome (36) to increase phage replicative fitness. Evolution experiments and screening for phage that escape from bacterial immune genes also readily identify point-mutations that occur at an appreciable rate in populations of similar size (35, 64, 65).

To experimentally validate our claims, we can model extant genomes by placing TA systems on mobile genetic elements (MGEs) integrated into laboratory model bacteria, like *E. coli*. In a co-culturing experiment, by introducing naïve bacteria, we can measure the direct HGT transfer of these MGEs across bacteria. By adding infecting phage to the system, we can measure the effect on MGE spread across the bacterial population, the potential acquisition of antitoxin genes from TA locus by the phage genome to evolve resistance, and the ability to maintain diverse genomes during co-evolution. While little known HGT mechanisms and processes constrain our model’s predictive power, the “Red Queen” dynamics of such competitive coevolution can be folded into a suitably generalized model and would partially stabilize bacterial/pathogen strains by reducing the effective rate of extinction. This would effectively push the bacterial pathogen system toward the endemic coexistence in Regime III of our phase diagram.

Finally, we note that our model of panimmunity is closely related to the multiple-islands-migration model in ref. 38. Genes and genotypes are analogous to strains and “islands,” respectively, and HGT plays a similar role as migration to stabilize the phage–bacteria dynamics. The spatial structure in their model is replaced with a genetic structure in ours, which allows for different applications.

To conclude, our study provides a mathematical illustration of the panimmunity hypothesis, showing how a sufficiently high rate of HGT can ensure persistence of a diverse repertoire of defense genes distributed across the pan-genome of bacteria and phages. Combinations of diverse defense genes in the same genome result in a high-dimensional genotype space, leading to the emergence of numerous unoccupied “microniches,” particularly when the interactions between defense and counterdefense genotypes are sparse. Our results imply that a relatively small number of thriving strains is sufficient to maintain diverse genes. These strains undergo rapid turnover among vast microniches, and the phage–bacteria arms race never ends.

## Supplementary Material

Appendix 01 (PDF)

## Data Availability

The code for simulations and plots can be found at https://github.com/Wenping-Cui/GeneTransfer (66). All other data are included in the article and/or *SI Appendix*.
