# Supplementary material for: A minimal model of panimmunity maintenance by horizontal gene transfer in the ecological dynamics of bacteria and phages
Source: Proc Natl Acad Sci U S A. 2025 Aug 1;122(31):e2417628122. doi: 10.1073/pnas.2417628122 (PMC12337353; doi:10.1073/pnas.2417628122)
Supplement: Supplementary file 1 — Appendix 01 (PDF) [file pnas.2417628122.sapp.pdf]

Supporting Information for

A model of pan-immunity maintenance by horizontal gene transfer in the ecological dynamics of bacteria and phages

Wenping Cui, Jemma M. Fendley, Sriram Srikant, and Boris Shraiman

Corresponding Wenping Cui, wenpingcui@kitp.ucsb.edu; Boris Shraiman, shraiman@ucsb.edu.

This PDF file includes:

Supporting text
Figs. S1 to S14
Table S1
SI References

Supporting Information Text

1. Summary of notation

We summarize the notation that appeared in the main text and supplemental information:

B	Bacteria genotype population size
V	Bacterial phage (virus) genotype population size
L	Number of genes. We always assume phages and bacteria have the same number of different genes L ranges from 25 to 50 and 15 to 25 for "doubleton" and "tripleton", respectively.
K	Number of genotypes, $K = \binom{L}{2} = L(L-1)/2$ for "doubleton"; for $K = \binom{L}{3}$ for "tripleton".
N_B, N_V	Total population sizes for the bacteria and phages. We set $N = N_B = N_V$ in the main text. N ranges from 10^5 to 10^8 in simulations.
s, ω	The bacterial intrinsic growth rate; the phage death rate. We set $s = \omega$ in the main text. We always set $s = 5 \times 10^{-3}$.
r_B, r_V	HGT rates for bacteria and phages. We set $r = r_B = r_V$ in the main text. r ranges from 10^{-8} to 10^{-2} in simulations.
n_G	Genotype abundance, no distinction for bacteria and phage
n_G^*	Average population size for one specific genotype, $n_G^* = N/K$. If the bacterium and phage are assumed to have different fixed points, we use n_B^* and n_V^* to represent them. n_G^* ranges from 10^2 to 10^5 in simulations.
n_g	Gene abundance, no distinction for defense and counter-defense systems
n_g^*	Average population size for one specific gene, $n_g^* = 2N/L$ for "doubleton"
ϕ	The infection rate. We parameterize $\phi = \frac{s}{\rho_n n_G^*}$, which ranges from 10^{-8} to 10^{-4} in our simulations.
β	Burst size. $\beta = \rho_s \rho_n$, which usually ranges from 1 to 100 in our simulations.
Θ	Effective temperature for Boltzmann (Gamma) distribution of genotype abundances
θ	Effective temperature for Boltzmann (Gamma) distribution of gene abundances
E	Lyapunov function, defined in eq. (3)
ρ_r	The ratio between the phage and bacteria HGT rates, $\rho_r = r_V/r_B$
ρ_s	The ratio between the phage's death rate and the bacterial intrinsic growth rate, $\rho_s = \omega/s$
ρ_n	The ratio between the total phage and bacterial population sizes, $\rho_n = N_V/N_B = n_V^*/n_B^*$ We typically range ρ_r, ρ_s and ρ_n from 0.1 to 10 in simulations.

We would like to note that we assume bacteria and phage have anti-symmetric parameters so that they share the same statistical properties. If the bacterial and phage have different parameters, we use notations X_B and X_V (X could be s, N, r).

2. Simulation

We must consider stochastic effects when the population size is finite. We use the τ-leaping method (1) to simulate eqs. (1) in the main text:

$$B_{ij}^{t+1} = \text{Poisson}\left(B_{ij}^t e^{s(1 - V_{ij}^t/n_V^*)}\right), \quad V_{ij}^{t+1} = \text{Poisson}\left(V_{ij}^t e^{\omega(B_{ij}^t/n_B^* - 1)}\right). \tag{1}$$

In the limit of strong HGT and $s \sim \omega \ll 1$ and notations in the main text, we can write the LV dynamics part of eqs. (1) into continuous equations,

$$\begin{aligned} \frac{dB_{ij}}{dt} &= sB_{ij}(1 - \frac{V_{ij}}{\rho_n n_G^*}) + \sqrt{B_{ij}}\eta_{ij}^B, \\ \frac{dV_{ij}}{dt} &= \rho_s s V_{ij}(\frac{B_{ij}}{n_G^*} - 1) + \sqrt{V_{ij}}\eta_{ij}^V. \end{aligned} \tag{2}$$

which is the LV dynamics of eqs. (1) in the main text with demographic noise. Here s and $\rho_s s$ are the host birth rate and the phage death rate. n_G^* and $\rho_n n_G^*$ are the host and phage characteristic population sizes which parameterize the infection rate and phage burst size; specifically $s/(\rho_n n_G^*)$ is the infection rate, and $\rho_s \rho_n$ is the phage's burst size. The last term is demographic noise, and η^X $(X = B, V)$ represents the unit white noise.

Algorithm 1 Stochastic clone-based algorithm

Set model parameters L, s, ρ_s, r_B, r_V, N_B, and N_V.
Initialize the population size $B_{ij}(t=0)$, $V_{ij}(t=0)$ for each strain (genotype) with n_B^* and n_V^*, respectively.
for t in $1:T$ **do**
    Selection process:
    Each bacterial strain replicates itself following a Poisson distribution with the rates $B_{ij}e^{s[1-V_{ij}^t/(\rho_n n_G^*)]+\gamma(1-\frac{\bar{N}_B}{N_B})}$, where $\bar{N}_B = \sum_{i<j} B_{ij}e^{s[1-V_{ij}^t/(\rho_n n_G^*)]}$. The term $\gamma(1-\frac{\bar{N}_B}{N_B})$ works as a regulator to constrain the total population approximately equal to N (2). We set $\gamma=0$ for no population constraint and $\gamma=\log 2$ for the hard constraint.
    The phage replication follows a similar procedure.
    Horizontal Gene transfer process:
    Select arbitrary $r_B N_B$ individuals from all the existing bacteria and $r_V N_V$ individuals from all the existing phages, both via uniform sampling.
    For the **inter-bacteria** HGT case, then randomly select (via uniform sampling) another $r_B N_B$ genes from the bacteria pool and replace one random gene from each of the selected $r_B N_B$ bacterial individuals with the selected genes. The **inter-phage** and **phage-bacteria** cases follow similar procedures.
    The **mean field approximation** case is equivalent to random mutations. To simulate this, we randomly mutate the selected genotype to an arbitrary genotype.
    Add the $r_B N_B$ new bacteria and $r_V N_V$ new phages back to the pool.

All simulations are conducted with Julia. The codes are available on GitHub at https:github.com/Wenping-Cui/GeneTransfer.

3. Canonical ensemble and Gamma distribution ansatz

In the deterministic limit, the antisymmetric Lotka-Volterra model for one bacterium-phage pair has the following conserved quantity, i.e., the Lyapunov function:

$$E = (B - n_G^* \log\frac{B}{n_G^*}) + (V - n_G^* \log\frac{V}{n_G^*}), \quad [3]$$

where the superscript * denotes the steady state.

Our system consists of many bacteria-phage pairs coupled by gene exchange processes. Employing the idea of the canonical ensemble for interacting Lotka-Volterra systems (3), the total Lyapunov function can be written as

$$E_{tot} = \sum_i (B_i + V_i - n_G^* \log\frac{B_i}{n_G^*} - n_G^* \log\frac{V_i}{n_G^*}). \quad [4]$$

When all genes coexist for a long time, we may assume it is in *thermal equilibrium* and E_{tot} is conserved. It has been shown that the phase space has a unit of $\log B_i \log V_i$ to secure Liouville's theorem (3, 4); thus, we can derive the Boltzmann distribution from the principle of maximum entropy:

$$\rho(B,V)\mathrm{dlog}B\,\mathrm{dlog}V \propto e^{-(B+V-n_G^*\log\frac{B}{n_G^*}-n_G^*\log\frac{V}{n_G^*})/\Theta}\mathrm{dlog}B\,\mathrm{dlog}V, \quad [5]$$

where Θ plays the role of an effective temperature. From the above expression, we can treat the bacterial and phage abundance distributions as independent and identical. We use n_G to represent either the bacterial or phage abundances and obtain

$$\rho(n_G) \propto \left(\frac{n_G}{n_G^*}\right)^{n_G^*/\Theta} n_G^{-1} e^{-n_G/\Theta}. \quad [6]$$

After normalization, this is equivalent to the *Gamma distribution*

$$\rho(n_G) = Gamma\ (n_G^*,\ \Theta) = \frac{n_G^{n_G^*/\Theta - 1} e^{-n_G/\Theta}}{\Theta^{n_G^*/\Theta}\Gamma(\frac{n_G^*}{\Theta})}, \quad [7]$$

where Θ is an unknown variable determined by system properties but can be evaluated numerically through

$$\Theta = \left\langle (n_G - n_G^*)^2 \right\rangle / n_G^*, \quad [8]$$

where the average $\langle \dots \rangle$ is taken over the whole time series in the thermodynamic equilibrium phase with all strains surviving.

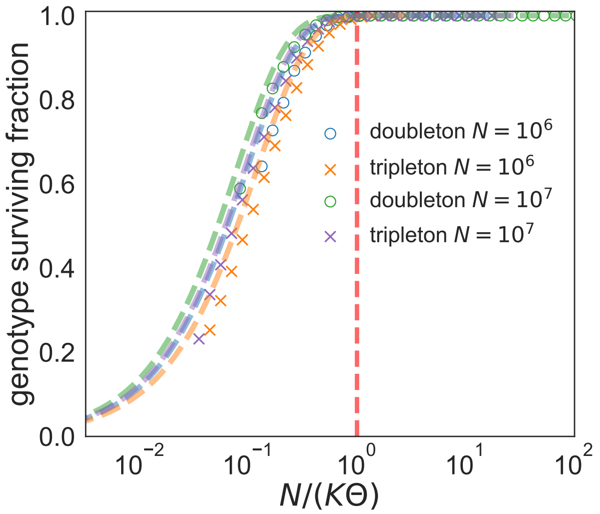

Fig. S1. Average surviving fraction of all genotypes over the whole time series. The vertical red dashed line is $N/(K\Theta) = 1$. The remaining dashed lines represent theoretical predictions from eq. 9, corresponding to the scatter points of the same colors. Here we obtain different Θ by adjusting the HGT rate r with $L = 40, s = 0.005$.

A. Surviving fraction. We can estimate the average surviving fraction of all possible genotypes over the time series by integrating the Gamma distribution from the natural cut-off 1 to infinity, yielding

$$f_s = \int_1^{+\infty} \frac{n_G^{n_G^*/\Theta-1} e^{-n_G/\Theta}}{\Theta^{n_G^*/\Theta}\Gamma(\frac{n_G^*}{\Theta})} dn_G = \Gamma\left(\frac{N}{K\Theta}, \frac{1}{\Theta}\right)/\Gamma(\frac{N}{K\Theta}). \tag{9}$$

We would like to note that here $\Gamma\left(\frac{N}{K\Theta}, \frac{1}{\Theta}\right)$ is the incomplete gamma function to avoid confusion with the Gamma distribution notation in the main text. Fig. S1 shows eq. (9) matches remarkably well with numerical simulation. Additionally, it reveals that the fraction of surviving genotypes begins to rapidly decline from 1 when the rate of the power law component of the Gamma distribution falls below 1, signifying the onset of Regime II, where genotype extinctions occur.

4. Stochastic LV model without horizontal gene transfer

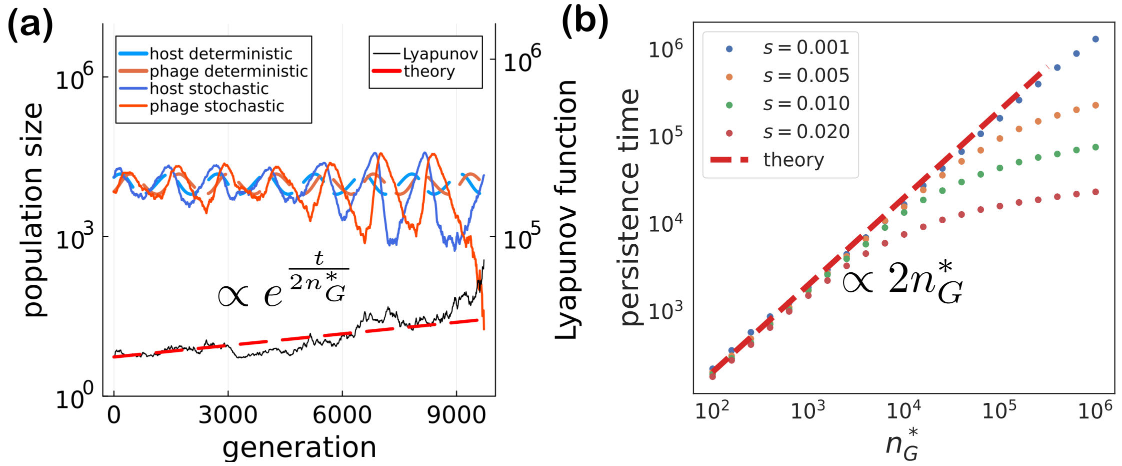

Fig. S2. Left y axis: deterministic and stochastic dynamics of one single pair of the bacteria-phage system. Right y axis: the corresponding Lyapunov function and the theoretical prediction. The steady state for the phage and bacteria is $n_G^* = 10^4$, and the intrinsic growth/death rate is $s = 5 \times 10^{-3}$; **(b)** Persistence time for different n_G^* and s. The data points are averaged over 1000 trials of simulation.

In the deterministic limit, the bacteria and phage dynamics form a prey-predator oscillator that can oscillate forever. When the population size is finite, the stochasticity will drive the oscillation amplitude to increase until either the bacteria or the phage goes extinct, as shown in Fig. S2a. In this section, we will derive the scaling of the extinction time.

When the gene exchange rate is negligible compared the typical selection rate,

$$r \ll s\,(1 - V/n_G^*) \sim s\,(B/n_G^* - 1) \ll 1, \tag{10}$$

we no longer consider HGT. Assuming the fitnesses for the bacterium and phage, $s\left(1-V/n_G^*\right)$ and $s\left(B/n_G^*-1\right)$, are much smaller than 1, we can consider the stochastic LV equations

$$\begin{aligned}\frac{dB}{dt}&=sB\left(1-V/n_G^*\right)+\sqrt{B}\eta_B,\\ \frac{dV}{dt}&=sV\left(B/n_G^*-1\right)+\sqrt{V}\eta_V,\end{aligned} \quad [11]$$

where η_B and η_V are white noises with $\langle\eta_B(t)\rangle=\langle\eta_V(t)\rangle=0$, $\langle\eta_B(t)\eta_V(t')\rangle=0$, $\langle\eta_B(t)\eta_B(t')\rangle=\delta(t-t')$, and $\langle\eta_V(t)\eta_V(t')\rangle=\delta(t-t')$.

Instead of studying the two-dimensional nonlinear LV dynamics, we can study its stability through the Lyapunov function (5) given by eq. (3). Substituting eq. (3) into the deterministic part of eqs. (11) shows that the Lyapunov function is conserved in the deterministic case, corresponding to a stable oscillator.

For the stochastic LV model, we can apply Itô's lemma (6),

$$\begin{aligned}dE&=\left(\frac{\partial E}{\partial B}\frac{dB}{dt}+\frac{\partial E}{\partial V}\frac{dV}{dt}\right)dt+\frac{1}{2}\left(\frac{\partial^2 E}{\partial B^2}B+\frac{\partial^2 E}{\partial V^2}V\right)dt+\sqrt{B}\frac{\partial E}{\partial B}d\eta_B+\sqrt{V}\frac{\partial E}{\partial V}d\eta_V\\ &=n_G^*\left(\frac{1}{2B}+\frac{1}{2V}\right)dt+\sqrt{\frac{(B-n_G^*)^2}{B}+\frac{(V-n_G^*)^2}{V}}d\eta\\ &=\frac{n_G^*(E+n_G^*\log\frac{B}{n_G^*}+n_G^*\log\frac{V}{n_G^*})}{2BV}dt+\sqrt{\frac{(B-n_G^*)^2}{B}+\frac{(V-n_G^*)^2}{V}}d\eta\end{aligned} \quad [12]$$

We can apply a linear-noise approximation around the steady state to approximate it as

$$dE\approx\left[\frac{E}{2n_G^*}-\frac{E(\Delta V+\Delta B)}{2(n_G^*)^2}+\frac{\Delta V+\Delta B}{2n_G^*}\right]dt+\sqrt{\frac{\Delta B^2+\Delta V^2}{n_G^*}}d\eta. \quad [13]$$

where $B=n_G^*+\Delta B$ and $V=n_G^*+\Delta V$. In the limit $n_G^*\gg\Delta V,\Delta B$, we can keep the zeroth order, and the Lyapunov function grows exponentially with time:

$$E(t)\approx E(0)e^{\frac{t}{2n_G^*}}. \quad [14]$$

We do not know the threshold of the Lyapunov function when the oscillator becomes unstable. However, from eq. (14), we can roughly estimate the typical persistence time of the oscillator to be about $2n_G^*$.

Fig. S2a shows one simulation trial with the initial condition around the steady state, and the Lyapunov function grows approximately like eq. (14). Fig. S2b compares the average persistence time with our theory, and $2n_G^*$ is a good estimation when the average clone size is below 10^5. This is surprising because our result is based on the linear-noise approximation, which is no longer valid when the system is far from the steady state. Luckily, the amplitude of the oscillator increases dramatically after the linear-noise approximation fails, so our approximation is not bad.

5. Regime III: stochastic oscillation with high HGT rates

A. Stochastic Lotka-Volterra equations with HGT. In this regime, the HGT rate is comparable to or larger than the fitness, i.e.,

$$s(-V/n_G^*+1)\sim s(B/n_G^*-1)\lesssim r\ll 1, \quad [15]$$

but both are still small compared with 1. We can write down the approximated Langevin equations:

$$\begin{aligned}\frac{dB}{dt}&=sB(-V/n_G^*+1)+rn_G^*+\sqrt{B}\eta_B,\\ \frac{dV}{dt}&=sV(B/n_G^*-1)+rn_G^*+\sqrt{V}\eta_V.\end{aligned} \quad [16]$$

The first term is the ordinary Lotka-Volterra dynamics. For the second term, we assume that the HGT process uniformly samples over the whole genotype space. This suggests HGT plays a similar role to migrations (7–9). The third term comes from the demographic noise.

Applying Itô's lemma (6), the stochastic differential equation of eq. (3) becomes

$$\begin{aligned}dE&=\left(\frac{\partial E}{\partial B}\frac{dB}{dt}+\frac{\partial E}{\partial V}\frac{dV}{dt}\right)dt+\frac{1}{2}\left(\frac{\partial^2 E}{\partial B^2}B+\frac{\partial^2 E}{\partial V^2}V\right)dt+\sqrt{B}\frac{\partial E}{\partial B}d\eta_B+\sqrt{V}\frac{\partial E}{\partial V}d\eta_V\\ &=\left(\frac{n_G^*}{2B}+\frac{n_G^*}{2V}+r\frac{n_G^*(B-n_G^*)}{B}+r\frac{n_G^*(V-n_G^*)}{V}\right)dt+\sqrt{\frac{(B-n_G^*)^2}{B}+\frac{(V-n_G^*)^2}{V}}d\eta.\end{aligned} \quad [17]$$

B. Self-consistency relations.

B.1. Solution of Θ and critical point. In this regime, we hope that the time average of the Lyapunov function does not change. We then evaluate the drift part averaged with the canonical ensemble

$$\int \rho(B)\rho(V)\left(\frac{n_G^*}{2B}+\frac{n_G^*}{2V}+r\frac{n_G^*(B-n_G^*)}{B}+r\frac{n_G^*(V-n_G^*)}{V}\right)dBdV=\frac{n_G^*}{(n_G^*-\Theta)}-\frac{2rn_G^*\Theta}{n_G^*-\Theta}=0. \quad [18]$$

where $\rho(B)$, $\rho(V)$ are Gamma distributions defined in eq. (6). The self-consistency relation gives

$$\Theta=\begin{cases}\frac{1}{2r}, & 2rn_G^*-1>0,\\ \text{no solution}, & 2rn_G^*-1\le 0.\end{cases} \quad [19]$$

Our analytical solution suggests there is a transition at $2rn_G^*=1$. The underlying reason is that the left tail of genotype Gamma distribution follows a power law with the exponent $2rn_G^*-1$. When the power exponent is below zero, $\rho(n_G)$ diverges at $n_G\to 0$, suggesting the phage and bacterium can go extinct, and only a fraction of genotypes are present in the system. Thus, the critical transition between Regime II and III happens at

$$r_c^G=\frac{1}{2n_G^*}=\frac{K}{2N}. \quad [20]$$

B.2. An alternative explanation from LV dynamics. We can see HGT processes help stabilize the system in the deterministic term in eq. (17). We want to ensure that the deterministic term is negative when the oscillation amplitude is large. Regarding the symmetry between B and V, we only consider the case $B\ll n_G^*$ and $V\gg n_G^*$ and ignore the phage part in the term. Then we obtain the stability condition: $\frac{n_G^*}{2B}-r\frac{(n_G^*)^2}{B}<0$, yielding

$$2rn_G^*-1>0, \quad [21]$$

which provides an alternative view of the critical transition at r_c^G, suggesting the predictions of r_c^G from canonical ensemble and stochastic LV dynamics are consistent with each other.

6. Regime II: boom-bust cycles with low HGT rates

When the HGT rate is weak, some phage or bacterial genotypes go extinct and take a long time to return to the ecosystem. In this regime, there are boom-bust cycles as in Fig. 3a, and the continuous stochastic Lotka-Volterra equation is no longer valid. Since the boom-bust cycles show that, most of the time, the phage does not impede the exponential growth of the susceptible bacterial strain until its establishment, we can apply a *Branching Process* analysis to this regime.

The rising bacterium has an approximately constant fitness s as its corresponding phage has a small population size. Fig. S3a shows that after the rising bacterium gets established, it will grow exponentially with rate s until the corresponding phage gets established.

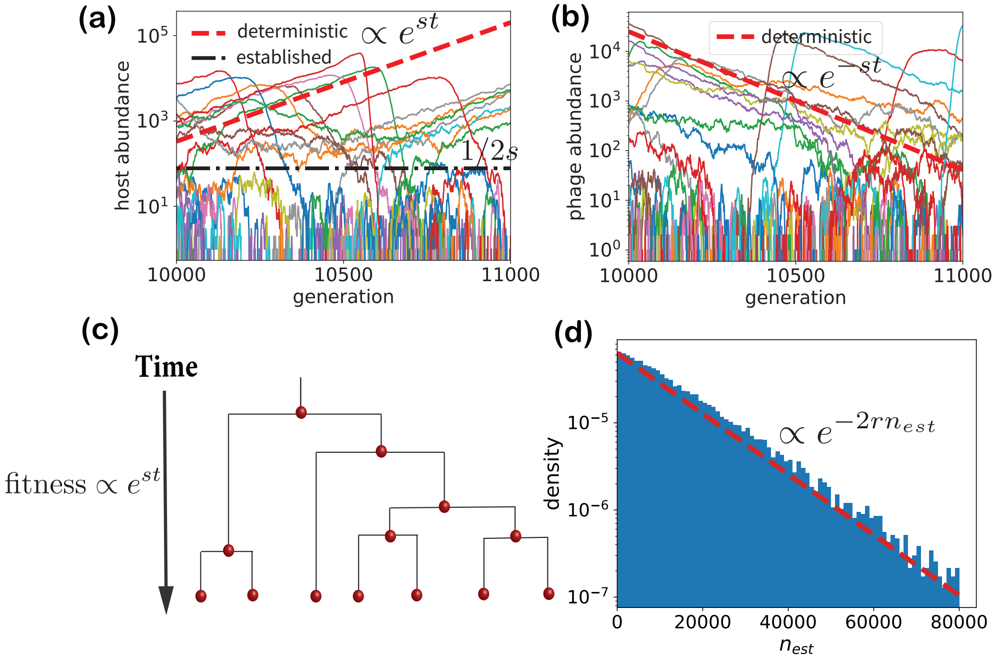

Fig. S3. **(a)** A snapshot of multiple-bacteria population dynamics. The black dotted-dash line is the typical established population size $\frac{1}{2s}$ as a boundary between stochastic fluctuation and deterministic growth $\propto e^{st}$ (the red dashed line). **(b)** A snapshot of multiple-phage population dynamics. The red dashed line shows the phage abundance almost decreased $\propto e^{-st}$ deterministically after dramatically booming. **(c)** A schematic illustrating a branching process of one strain of phages with its fitness increasing exponentially in time as its susceptible bacterium follows exponential growth. **(d)** The distribution of the bacterium abundance at the moment when the phage gets established and starts booming follows our theoretical prediction: an exponential distribution $\propto e^{-2rn_{est}}$.

A. Establishment (fixation) probability in a changing environment. Assuming the bacterium starts to grow exponentially at $t = 0$ with some established population size n_0, the phage's fitness x follows

$$x(t) = s\left(\frac{n_0}{n_G^*}e^{st} - 1\right). \qquad [22]$$

Eq. (22) shows the phage only has a positive fitness after the bacterium exceeds the mean population size, and thus we are only interested in the time after the bacterium population size exceeds n_G^*. We set the time when the bacterium population size equals n_G^* as zero. We want to answer the question: after a duration τ, there are corresponding phages reborn from HGT; what is the probability that they get established? This can be solved by a branching process in a changing environment (10) (also see the scheme at Fig. S3c).

We rewrite the phage's fitness into

$$x(\tau) = s(e^{s\tau} - 1), \qquad [23]$$

where $\tau = 0$, as we defined, is when the bacterium population exceeds its mean abundance.

We assume the extinction probability after t generations for a phage sampled at a given time τ follows a branching process with two offspring:

$$w(t|\tau) = [1 - (x(\tau) + 2)\Delta t]w(t - \Delta t|\tau + \Delta t) + \Delta t + (1 + x(\tau))\Delta t w(t - \Delta t|\tau + \Delta t)^2. \qquad [24]$$

It is easier to use the variable x instead of τ, where x is the fitness at time τ,

$$w(t|x) = [1 - (x + 2)\Delta t]w(t - \Delta t, x + s(x + s)\Delta t) + \Delta t + (1 + x)\Delta t w(t - \Delta t, x + s(x + s)\Delta t)^2. \qquad [25]$$

In the continuum limit,

$$\frac{\partial w(t|x)}{\partial t} - s(x + s)\frac{\partial w(t|x)}{\partial x} = 1 - (x + 2)w(t, x) + (1 + x)w(t|x)^2. \qquad [26]$$

We can rewrite the above equation into the survival probability $\phi(t, x) = 1 - w(t|x)$,

$$\frac{\partial \phi(t, x)}{\partial t} - s(x + s)\frac{\partial \phi(t, x)}{\partial x} = x\phi(t, x) - (1 + x)\phi(t, x)^2. \qquad [27]$$

We are interested in the establishment probability, which is equivalent to the survival probability in the long, but finite, time limit. In this limit, $\phi(t, x)$ is not sensitive to time and can be written as $\phi(x)$,

$$-s(x + s)\frac{\partial \phi(x)}{\partial x} = x\phi(x) - (1 + x)\phi(x)^2. \qquad [28]$$

In the limit of $sx \ll 1$ and $x \gg s$,

$$\phi(x) \approx \frac{x}{1+x} \approx \frac{se^{s\tau}}{1+se^{s\tau}}. \qquad [29]$$

In deriving the survival probability, we assumed there were two offspring in the birth-death process. Our simulation uses a Poisson process to sample the number of offspring, (see Sec. B), which contributes a factor of 2. After correction, it becomes

$$\phi(\tau) \approx \frac{2se^{s\tau}}{1+2se^{s\tau}}. \qquad [30]$$

B. Sampling from HGT. We assume the phage sampled from HGT follows a Poisson process with a constant event rate $\frac{r}{K}$ per individual per generation per strain. In principle, the HGT process depends on the time-varying gene abundance. However, Fig. S12 shows the gene dynamics, as the sum of $L-1$ genotype dynamics, have much smaller fluctuations and are also weakly correlated with the genotype dynamics, so we can take the average value and assume the event rate is a constant. The probability for sampling n phages of a specific genotype is given as

$$P(n) = \frac{e^{-\frac{rN}{K}}}{n!}\left(\frac{rN}{K}\right)^n. \qquad [31]$$

We must consider that the establishment probability varies at different generations. The probability of k established phages born at generation τ is given by

$$\begin{aligned} P(k,\tau) &= \sum_{n=k}^{\infty} \frac{e^{-\frac{rN}{K}}}{n!}\left(\frac{rN}{K}\right)^n \binom{n}{k} \phi(\tau)^k (1-\phi(\tau))^{n-k} \\ &= \frac{e^{-\frac{rN\phi(t)}{K}}}{k!}\left(\frac{rN\phi(t)}{K}\right)^k \sum_{n=k}^{\infty} \frac{e^{-\frac{rN}{K}+\frac{rN\phi(t)}{K}}}{(n-k)!}\left(\frac{rN}{K}-\frac{rN\phi(t)}{K}\right)^{n-k} \\ &= \frac{e^{-\frac{rN\phi(t)}{K}}}{k!}\left(\frac{rN\phi(t)}{K}\right)^k, \end{aligned} \qquad [32]$$

which is an inhomogeneous Poisson distribution (11, 12).

We are interested in when the first phage becomes established between T and $T+\Delta T$. In our simulation, we set $\Delta T = 1$, and the survival probability after exactly T generations follows an exponential distribution,

$$P(T) = \frac{rN}{K}\phi(T)e^{-\frac{rN}{K}\int_0^T \phi(\tau)\mathrm{d}\tau}. \qquad [33]$$

We can evaluate its exponential part by

$$\frac{rN}{K}\int_0^T \phi(\tau)\mathrm{d}\tau = \frac{rN}{K}\int_0^T \frac{2se^{s\tau}}{1+2se^{s\tau}}\mathrm{d}\tau = \frac{rN}{Ks}\log\left(\frac{1+2se^{sT}}{1+2s}\right) \approx 2rn_G^* e^{sT}. \qquad [34]$$

Here we employ the fact that a typical establishment time T obeys $se^{sT} \ll 1$ because the phage fitness is small.

We can write down its approximated probability distribution and consider the normalization

$$P(T) \approx 2srn_G^* e^{2rn_G^* + sT - 2rn_G^* e^{sT}}. \qquad [35]$$

Interestingly, this is the half-truncated *Gumbel distribution.*

We are interested in the bacterium population size when the first phage gets established; it follows (see Sec. 10)

$$Q(n_G) = \frac{1}{sn_G}P\left(\frac{1}{s}\log\frac{n_G}{n_G^*}\right) = 2re^{-2r(n_G - n_G^*)}, \quad n_G^* \le n_G < \infty. \qquad [36]$$

We examine the above equation with numerical simulations, and it fits well as shown in Fig. S3d.

C. Consistency with canonical ensemble. To be self-consistent with the exponential tail of the Gamma distribution in eq. (7), the effective temperature is given by

$$\Theta \approx \frac{1}{2r}. \qquad [37]$$

A question is raised as to why eq. (36) follows the exponential distribution instead of the Gamma distribution. This is because we draw the histogram from the whole time series, which can be viewed as the sum of many independent and identical exponential distributions, naturally yielding the Gamma distribution. Our analysis matches well with simulation, as shown in Fig. 4 in the main text.

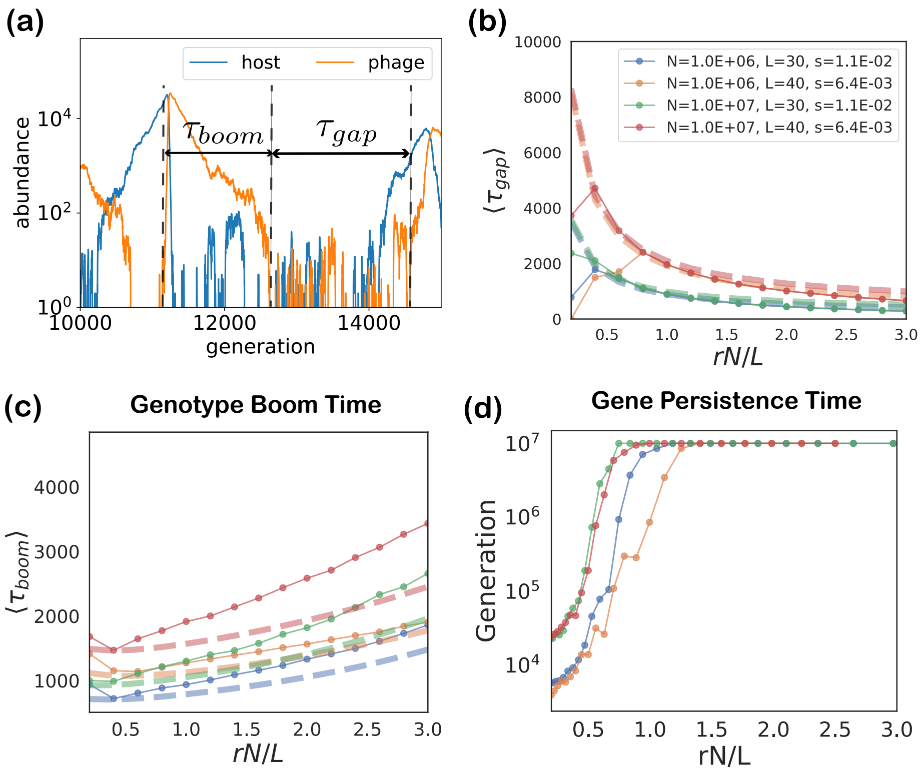

Fig. S4. **(a)** Scheme for the definition of the gap time τ_{gap} between two booms of phage and the phage booming time τ_{boom} in the boom-bust dynamics. **(b, c)** Comparison of the averaged τ_{gap} and τ_{boom} from simulation and our theoretical predictions (dashed lines). **(d)** Gene survival time for different L and N with a simulation time limit of 10^7 generations.

D. Booming duration and time gap between consecutive booms. It is interesting to investigate properties of the boom-bust cycles: the boom duration τ_{boom}, defined as the time elapsed when the bacterium gets established ($n_{est} \sim \frac{1}{2s}$), reaches the peak ($n_G \sim \frac{1}{2r}$) and then dies out, $n_G = 0$; and the gap time between two peaks, τ_{gap}, shown in Fig. S4a. Because of time-reversal symmetry (perfect anti-symmetry), τ_{gap} and τ_{boom} are identical for phages and bacteria.

Considering the bacterium, we can estimate the booming duration by the following equation:

$$\tau_{boom} = \frac{1}{s} \log \frac{s}{r} + \frac{4rn_G^*}{s(1-2rn_G^*)} \log \frac{1}{2r}. \qquad [38]$$

The first term describes the exponential growth time from the established population size to the typical boom size. The second term describes two sequential processes: the phage reaches its boom size, and then, the bacterium drops to zero. We assume the two processes have the same typical time scale, contributing a factor of 2.

τ_{gap}, the time between when the bacterium/phage goes extinct and when it gets established, can be written as

$$\tau_{gap} = \frac{1}{2s}\left(\frac{1}{rn_G^*} + \log \frac{s}{r}\right). \qquad [39]$$

The first term describes the typical time for a bacterial strain with a survival probability $2s$ and a HGT rate rN/K to get established (11). The second term results from the correction that the bacterial boom is always followed by the phage boom, and the bacterium cannot get established when the phage is in the boom phase.

In the simulation, τ_{boom} for the bacterium is underestimated. This is because the bacterium has a higher chance to undergo continuous booms after the phage dies, but the phage dies immediately after the bacterium dies. In Fig. S4bc, we show our analytical estimation agrees well with the simulation.

Fig. S4d gives a higher resolution of r near the transition between Regime I and II. $rN/L = 1$ is the critical boundary given by eqs. (6) in the main text. It shows when passing r_c^g, i.e., $rN/L > 1$, the strain boom time does not vary much, but the gene persistence time can increase dramatically, much faster than exponential growth.

E. Mean population size without the population size constraint. In Regime III, the prey-predator oscillator oscillates around the steady state n_G^*, causing the mean population size over the time series to also be n_G^*. However, in the boom-bust dynamics, for instance, the bacterium undergoes exponential growth and catastrophic recession, which does not have a steady state. We choose a hard constraint for the total population size and hence the average must be equal to n_G^*.

The reader may be curious about the average clone size of each strain if we remove the hard constraint about the total population size. The gap period between two booms is given by

$$\tau_{gap} \approx \frac{1}{2srn_G^*}, \tag{40}$$

which leads to

$$\langle n \rangle = \frac{\int_0^{\frac{1}{s}\log\frac{s}{r}} dt \frac{1}{2s} e^{st}}{\frac{1}{s}\log\frac{s}{r} + \tau_{gap}} = \frac{\frac{1}{2sr} - \frac{1}{2s^2}}{\frac{1}{s}\log\frac{s}{r} + \frac{1}{2srn_G^*}} \approx n_G^*. \tag{41}$$

The above equation is a rough estimation. It needs careful treatment when the phages and bacteria are not perfectly anti-symmetric.

7. Different mechanisms of horizontal gene transfer

For the gene abundance distribution, it is helpful to normalize the population size to its fraction:

$$\begin{aligned} \frac{dP_{ij}}{dt} &= sKP_{ij}\left(\frac{1}{K} - Q_{ij}\right) + J_{ij}^P + \sqrt{\frac{P_{ij}}{N}}\eta_P, \\ \frac{dQ_{ij}}{dt} &= sKQ_{ij}\left(P_{ij} - \frac{1}{K}\right) + J_{ij}^Q + \sqrt{\frac{Q_{ij}}{N}}\eta_Q, \end{aligned} \tag{42}$$

where $P_{ij} = \frac{1}{N}B_{ij}$, $Q_{ij} = \frac{1}{N}V_{ij}$ are the fraction of bacterium/phage genotypes carrying genes i, j so that the average of the off-diagonal elements $\langle P_{ij} \rangle = \langle Q_{ij} \rangle = 1/K$. We set $P_{ii} = Q_{ii} = 0$, $P_{ij} = P_{ji}$, and $Q_{ij} = Q_{ji}$ as each individual carries two different genes, and the order of genes does not affect the genotype. In other words. P_{ij} and Q_{ij} are symmetric matrices with diagonal elements equal to zero. J_{ij}^X, represents the influx of clones for a specific genotype X_{ij}, and their forms depend on the detailed HGT processes.

We would like to emphasize that the scaling of θ and Θ is also changed after normalization of the population size. As a result, we define $\bar{\theta}$ and $\bar{\Theta}$ for the normalized genotype and gene abundances, which obey

$$\theta = N\bar{\theta}, \quad \Theta = N\bar{\Theta}.$$

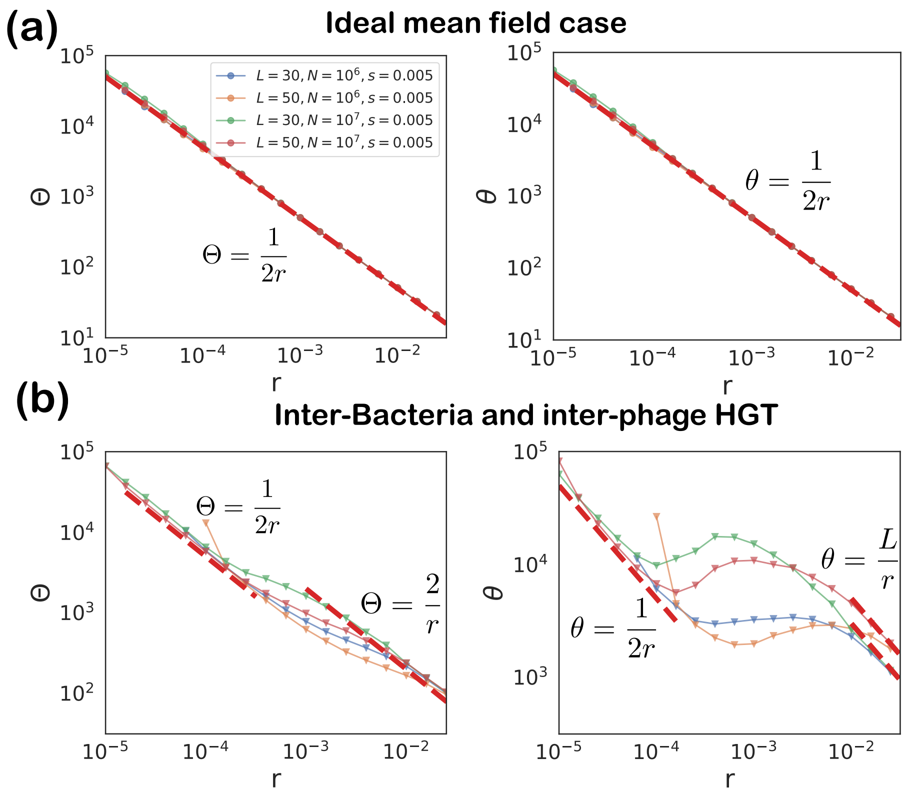

Fig. S5. Effective genotype and gene temperature for **(a)** the ideal mean field case, **(b)** inter-bacteria and inter-phage HGT processes. The red dashed lines are theoretical predictions.

A. Ideal mean-field case. In this ideal case, we assume the rate of genotype generation is constant so that the clone influx is also constant. The dynamics are

$$\begin{aligned}\frac{dP_{ij}}{dt} &= sKP_{ij}\left(\frac{1}{K}-Q_{ij}\right)+\frac{r}{K}+\sqrt{\frac{P_{ij}}{N}}\eta_P,\\ \frac{dQ_{ij}}{dt} &= sKQ_{ij}\left(P_{ij}-\frac{1}{K}\right)+\frac{r}{K}+\sqrt{\frac{Q_{ij}}{N}}\eta_Q.\end{aligned} \quad [43]$$

We are interested in the marginal probability of genes: $p_i=\sum_j P_{ij}$ and $q_i=\sum_j Q_{ij}$. We would like to note that

$$\sum_i p_i = \sum_i q_i = 2, \quad [44]$$

as each individual carries two genes, so it is not normalized. The influx for one specific gene is

$$j_i^p = \sum_j J_{ij}^P = \frac{2r}{L},\quad j_i^q = \sum_j j_{ij}^q = \frac{2r}{L}, \quad [45]$$

which are also constant in this ideal case.

The dynamics of p_i, q_i become

$$\begin{aligned}\frac{dp_i}{dt} &\approx sp_i - sK\sum_j P_{ij}Q_{ij}+\frac{2r}{L}+\sqrt{\frac{p_i}{N}}\eta_p,\\ \frac{dq_i}{dt} &\approx sK\sum_j P_{ij}Q_{ij}-sq_i+\frac{2r}{L}+\sqrt{\frac{q_i}{N}}\eta_q.\end{aligned} \quad [46]$$

We can evaluate the second term, $\sum_j P_{ij}Q_{ij}$, by assuming P_{ij} and Q_{ij} are weakly correlated:

$$\sum_j P_{ij}Q_{ij} \approx \frac{1}{L}p_i q_i. \quad [47]$$

Combining the above relations, we have

$$\begin{aligned}\frac{dp_i}{dt} &\approx \frac{Ls}{2}p_i\left(\frac{2}{L}-q_i\right)+\frac{2r}{L}+\sqrt{\frac{p_i}{N}}\eta_p,\\ \frac{dq_i}{dt} &\approx \frac{Ls}{2}q_i\left(p_i-\frac{2}{L}\right)+\frac{2r}{L}+\sqrt{\frac{q_i}{N}}\eta_q.\end{aligned} \quad [48]$$

Similar to eq. (17), we can calculate the effective temperature of the Gamma distribution for p_i, q_i, and then rescale back to its population size. This gives

$$\theta = \frac{1}{2r}. \quad [49]$$

B. Inter-bacteria and phage-bacteria HGT. In this case, we take the phage-bacteria and inter-bacteria HGT into consideration, and the inflow HGT terms in eq. (42) become

$$\begin{aligned}J_{ij}^p =& \frac{r}{4}\sum_{j'\neq j}P_{ij'}p_j+\frac{r}{4}\sum_{i'\neq i}P_{ij'}p_i+\frac{r}{2}P_{ij}(p_i+p_j)\\ =& \frac{r}{4}(p_i-P_{ij})p_j+\frac{r}{4}(p_j-P_{ij})p_i+\frac{r}{2}P_{ij}(p_i+p_j)\\ =& \frac{r}{2}p_ip_j+\frac{r}{4}P_{ij}(p_i+p_j).\end{aligned}$$

Looking at the the first line of the above equation, $rP_{ij'}p_j/2$ and $rP_{ij'}p_i/2$ describe the probability that one strain with one gene different, picks up gene i and transforms into genotype ij, noting that $p_i/2$ results from the normalization of p_i. It is then divided by another factor of 2 since we do not count the order of two genes. $rP_{ij}(p_i/2+p_j/2)$ describes the probability that one genotype stays the same after the HGT. Similarly, we have

$$J_{ij}^q = \frac{r}{4}\sum_{j'}Q_{ij'}p_j+\frac{r}{4}\sum_{i'}Q_{ij'}p_i+\frac{r}{2}Q_{ij}(p_i+p_j) = \frac{r}{4}q_ip_j+\frac{r}{4}q_jp_i+\frac{r}{4}Q_{ij}(p_i+p_j).$$

We can write the gene dynamics as

$$\begin{aligned}\frac{dp_i}{dt} &= \frac{Ls}{2}p_i\left(\frac{2}{L}-q_i\right)+\frac{r}{2}p_i\sum_j p_j+\frac{r}{4}p_i^2+\frac{r}{4}\sum_j P_{ij}p_j+\sqrt{\frac{p_i}{N}}\eta_p,\\ \frac{dq_i}{dt} &= \frac{Ls}{2}q_i\left(p_i-\frac{2}{L}\right)+\frac{r}{4}q_i\sum_j p_j+\frac{r}{4}p_i\sum_j q_j+\frac{r}{4}p_iq_i+\frac{r}{4}\sum_j Q_{ij}p_j+\sqrt{\frac{q_i}{N}}\eta_q.\end{aligned} \quad [50]$$

Utilizing $\sum_{j\neq i} p_j = 2 - p_i$ and $\sum_{j\neq i} q_j = 2 - q_i$, it can be reduced to

$$\begin{aligned}\frac{dp_i}{dt} &= \frac{Ls}{2}p_i\left(\frac{2}{L}-q_i\right)+\frac{r}{4}\sum_j P_{ij}p_j-\frac{r}{4}p_i^2+rp_i+\sqrt{\frac{p_i}{N}}\eta_p,\\ \frac{dq_i}{dt} &= \frac{Ls}{2}q_i\left(p_i-\frac{2}{L}\right)+\frac{r}{2}\left(p_i-q_i\right)+\frac{r}{4}\sum_j Q_{ij}p_j-\frac{r}{4}p_iq_i+rq_i+\sqrt{\frac{q_i}{N}}\eta_q.\end{aligned} \quad [51]$$

B.1. Decoupling of gene and genotype dynamics. Fig. S12 suggests the different gene and genotype dynamics are weakly correlated. Hence we can average the gene dynamics out:

$$\sum_j P_{ij}p_j \approx \frac{2}{L}p_i, \quad \sum_j Q_{ij}p_j \approx \frac{2}{L}q_i. \quad [52]$$

The mean-field gene dynamics become

$$\begin{aligned}\frac{dp_i}{dt} &= \frac{Ls}{2}p_i\left(\frac{2}{L}-q_i\right)+\frac{r}{4}p_i(\frac{2}{L}-p_i)+rp_i+\sqrt{\frac{p_i}{N}}\eta_p,\\ \frac{dq_i}{dt} &= \frac{Ls}{2}q_i\left(p_i-\frac{2}{L}\right)+\frac{r}{2}\left(p_i-q_i\right)+\frac{r}{4}q_i(\frac{2}{L}-p_i)+rq_i+\sqrt{\frac{q_i}{N}}\eta_q.\end{aligned} \quad [53]$$

With $p^* = q^* = \frac{2}{L}$, similar to eq. (17), we can write down the dynamics of the Lyapunov function for the gene dynamics as:

$$\begin{aligned}dE =& \left(\frac{p^*}{2Np_i}+\frac{p^*}{2Nq_i}-\frac{r}{4}(p_i-p^*)^2-\frac{r}{2q_i}(q^*-q_i)^2\right)dt\\ &+(\frac{r}{2q_i}-\frac{r}{4})(p_i-p^*)(q_i-q^*)dt+r(p_i-p^*)dt+r(q_i-q^*)dt+\sqrt{\frac{(p_i-p^*)^2}{Np_i}+\frac{(q_i-p^*)^2}{Nq_i}}d\eta.\end{aligned} \quad [54]$$

The self-consistency relation yields

$$\begin{aligned}&\int \rho(p_i)\rho_(q_i)\left[\frac{p^*}{2Np_i}+\frac{p^*}{2Nq_i}-\frac{r}{4}(p_i-p^*)^2-\frac{r}{2q_i}(q^*-q_i)^2\right]dq_idp_i\\ =&p^*\left[\frac{1}{N(p^*-\bar{\theta})}-\frac{r}{4}\bar{\theta}-\frac{r\bar{\theta}}{2(p^*-\bar{\theta})}\right]=0.\end{aligned} \quad [55]$$

In the limit $rN \gg 1$, it gives

$$\theta = N\bar{\theta} = N\left[\frac{L+1}{L}-\sqrt{\left(\frac{L+1}{L}\right)^2-\frac{4}{rN}}\right]\approx\frac{2}{r}. \quad [56]$$

C. Inter-bacteria and inter-phage HGT. Similar to the phage-bacteria HGT case, we can write down the mean-field gene dynamics as

$$\begin{aligned}\frac{dp_i}{dt} &= \frac{2s}{L}p_i\left(\frac{2}{L}-q_i\right)+\frac{r}{4}p_i(\frac{2}{L}-p_i)+rp_i+\sqrt{\frac{p_i}{N}}\eta_p,\\ \frac{dq_i}{dt} &= \frac{2s}{L}q_i\left(p_i-\frac{2}{L}\right)+\frac{r}{4}q_i(\frac{2}{L}-q_i)+rq_i+\sqrt{\frac{q_i}{N}}\eta_q.\end{aligned} \quad [57]$$

The dynamics of the Lyapunov function are characterized by

$$\begin{aligned}dE =& \left(\frac{p^*}{2Np_i}+\frac{q^*}{2Nq_i}-\frac{r}{4}(p_i-p^*)^2-\frac{r}{4}(q_i-q^*)^2\right)dt\\ &+r(p_i-p^*)dt+r(q_i-q^*)dt+\sqrt{\frac{(p_i-p^*)^2}{Np_i}+\frac{(q_i-p^*)^2}{Nq_i}}d\eta.\end{aligned} \quad [58]$$

The self-consistency relation yields

$$\int \rho(p_i)\rho_(q_i)\left[\frac{p^*}{2Np_i}+\frac{q^*}{2Nq_i}-\frac{r}{4}(p_i-p^*)^2-\frac{r}{4}(q_i-q^*)^2\right]dq_idp_i=\frac{p^*}{N(p^*-\bar{\theta})}-\frac{r}{2}p^*\bar{\theta}=0, \quad [59]$$

which gives

$$\theta = N\bar{\theta} = N\left(\frac{1}{L}-\sqrt{\frac{1}{L^2}-\frac{2}{Nr}}\right)\approx\frac{L}{r}. \quad [60]$$

C.1. Effective genotype temperature. The above result shows the effective gene temperature is proportional to L, much larger than the phage-bacteria case. This strong gene-gene correlation leads to a correction to Θ, which is challenging to evaluate from the genotype dynamics as we can no longer decouple the gene and genotype dynamics.

Thanks to the quasi-linkage equilibrium in the strong HGT regime (13), we can approximate the genotype fraction by the product of its marginal gene fraction, $P_{ij} \propto p_i p_j, Q_{ij} \propto q_i q_j$ and obtain that P_{ij}, Q_{ij} follow a PDF:

$$\rho(x) = \frac{2x^{rN/L^2-1} K_0(2rN\sqrt{x}/L)}{(\frac{L}{rN})^{2rN/L^2}[\Gamma(rN/L^2)]^2}, \qquad [61]$$

where x represents either P_{ij} or Q_{ij}, and K_n is the modified Bessel function of the second kind. The above PDF has mean $1/L^2$ and variance $\frac{L^2+2Nr}{N^2L^2r^2}$. Then we can use eq. (61) to roughly estimate Θ for the Gamma distribution, which gives

$$\Theta \approx \frac{L^2 + 2Nr}{Nr^2} \approx \frac{2}{r}. \qquad [62]$$

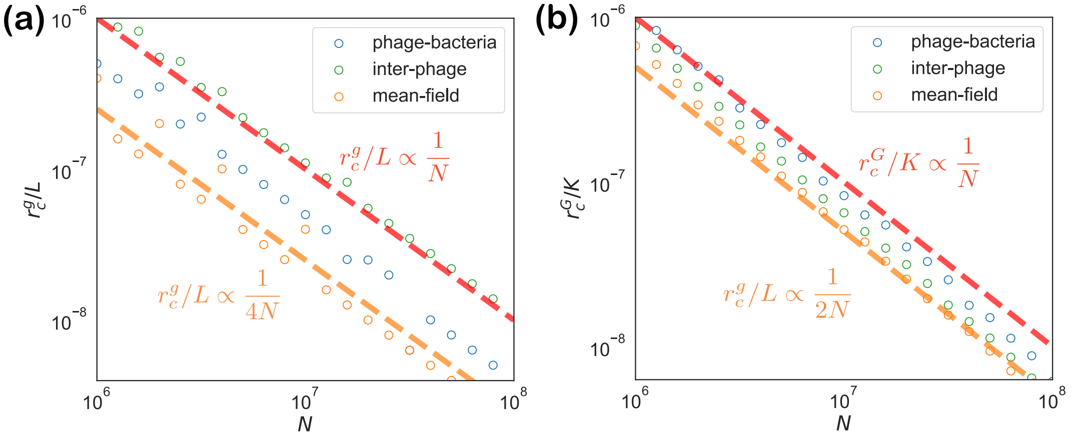

Fig. S6. Comparison of minimal HGT rates for (a) gene coexistence and (b) genotype coexistence for different types of HGT: inter-bacteria and phage-bacteria HGT, inter-bacteria and inter-phage HGT. We also conduct simulations with constant rates of genotype generation from the mean-field approximation for comparison. As expected, our mean field theoretical prediction (the orange lines) matches perfectly with the simulation with constant rates of genotype generation from mean field estimation, but falls off with a factor within order 1 for the two different HGT processes (the red lines are theoretical predictions dropping the prefactor).

D. Summary. We summarize the analytical results in Table S1 and show the comparison with numerical simulations in Fig. 4ab in the main text and Fig. S5. The scaling of Θ and θ shows different implementations of the HGT processes do not affect our criteria eqs. (7) much (see Fig. S6). We get the critical r by comparing the extinction time of the first gene by running a simulation with a large threshold $T = 250000$. We would like to note that for the ideal mean-field case, the extinct gene can always come back in the future, so the scaling of r_c^g appearing in Fig. S6 works as an ideal case to compare with other cases and examine our theory.

HGT	Boom-bust cycles		Stochastic oscillator	
	Θ	θ	Θ	θ
mean-field	$1/2r$	$1/2r$	$1/2r$	$1/2r$
inter-bacteria/ phage-bacteria	$1/2r$	$1/2r$	$1/2r$	$2/r$
inter-bacteria/ inter-phage	$1/2r$	$1/2r$	$2/r$	L/r

Table S1. Summary of effective temperature for different types of HGT.

8. General parametrization

A. Distinct parameters for bacteria and phages. In the main text, we set the ratios: $\rho_r = r_V/r_B$, $\rho_s = \omega/s$, and $\rho_n = n_V^*/n_B^*$ all equal to 1 to reduce the parameter space. In nature we often anticipate the phage to have a larger clone size $\rho_n > 1$, a higher HGT rate $\rho_r > 1$, and for the burst size, proportional to ρ_s, to be also larger than 1. Let's investigate how they affect the effective temperature in Regime III first.

A.1. Regime III. In this regime, the dynamics become

$$\frac{dB}{dt} = sB\left(1 - \frac{V}{\rho_n n_G^*}\right) + r n_G^* + \sqrt{B}\eta_B,$$
$$\frac{dV}{dt} = \rho_s s V(B/n_G^* - 1) + \rho_r r b n_G^* + \sqrt{V}\eta_V, \quad [63]$$

where $r = r_B$, and $N = N_B$.

The Lyapunov function becomes

$$E = \rho_n(B - n_G^* \log \frac{B}{n_G^*}) + \frac{1}{\rho_s}(V - \rho_n n_G^* \log \frac{V}{\rho_n n_G^*}). \quad [64]$$

We define the effective temperature T, and the bacterium, phage abundances follow

$$B \sim Gamma\,(n_G^*,\ \Theta_B = T/\rho_n)\,, \quad V \sim Gamma\,(-\rho_n n_G^*,\ \Theta_V = \rho_s T)\,. \quad [65]$$

Repeating the above steps, the self-consistency equation is

$$\left\langle \frac{\rho_n n_G^*}{2B} + \frac{\rho_n n_G^*}{2\rho_s V} + \rho_n r \frac{n_G^*(B - n_G^*)}{B} + \rho_r r \frac{\rho_n n_G^*(V - \rho_n n_G^*)}{\rho_s V} \right\rangle_{\rho(B),\rho(V)} = 0. \quad [66]$$

After taking the average, it becomes

$$\frac{\rho_s(\rho_n - 2rT)}{\rho_n n_G^* - T} + \frac{(1 - 2\rho_r\rho_s rT)}{\rho_n n_G^* - \rho_s T} = 0. \quad [67]$$

We drop the nonphysical solution, and the other solution of T is a complicated expression:

$$T = \frac{\rho_n(\rho_s^2 + 2(1+\rho_r)\rho_s n_G^* r + 1) - \sqrt{\rho_n^2\left(\rho_s^2 + 2(\rho_r+1)\rho_s n_G^* r + 1\right)^2 - 8\rho_n\rho_s(\rho_s\rho_n + 1)n_G^* r(\rho_r + \rho_s)}}{4\rho_s r(\rho_r + \rho_s)}. \quad [68]$$

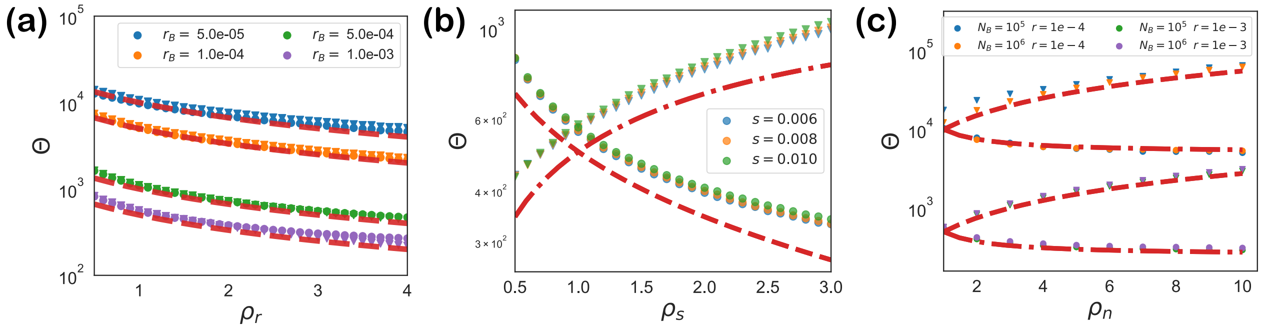

Fig. S7. Effective genotype temperature Θ for bacteria and phages with **(a)** different HGT rates, **(b)** interaction strength and **(c)** population size. The dashed lines and dash-dotted lines are predictions from the theory for bacteria and phages respectively. The scatter points are simulation data with $L = 40$, $r = 10^{-3}$, $s = 5 \times 10^{-3}$, and $N = 10^6$ unless specified. Circular and triangular markers represent bacteria and phages, respectively.

If we only change one pair of parameters and keep the other two identical, we find simpler expressions:

- Different gene exchange rate r:

$$\Theta_B = \Theta_V = \frac{1}{(1+\rho_r)r}. \quad [69]$$

- Different N:

$$\Theta_B = \frac{T}{\rho_n} = \frac{1+\rho_n}{4br}, \quad \Theta_V = T = \frac{1+\rho_n}{4r}. \quad [70]$$

- Different s :

$$\Theta_B = \frac{1 + 4\rho_s n_G^* r + \rho_s^2 - \sqrt{1 + 2\rho_s^2 + 16\rho_s^2 n_G^* r(n_G^* r - 1) + \rho_s^4}}{4\rho_s(\rho_s+1)r},$$
$$\Theta_V = \frac{1 + 4\rho_s n_G^* r + \rho_s^2 - \sqrt{1 + 2\rho_s^2 + 16\rho_s^2 n_G^* r(n_G^* r - 1) + \rho_s^4}}{4(\rho_s+1)r}. \quad [71]$$

We examine the above results with simulation in Fig. S7.

A.2. Regime II. We rewrite the phage's fitness, eq. 22, into

$$x(\tau) = \rho_s s(e^{s\tau} - 1). \quad [72]$$

The survival probability is given by

$$\phi(x) \approx \frac{x}{1+x} \approx \frac{\rho_s s e^{s\tau}}{1+\rho_s s e^{s\tau}}. \quad [73]$$

The first passage time for the phage to get established is given by

$$P(T) \approx 2s\rho_r\rho_s\rho_n r n_G^* e^{2\rho_r\rho_s\rho_n r n_G^* + sT - 2\rho_r\rho_s\rho_n r n_G^* e^{sT}}. \quad [74]$$

We are interested in the bacterium population size when the first phage gets established;

$$Q(B) = \frac{1}{sB}P(\frac{1}{s}\log\frac{B}{n_G^*}) = 2\rho_r\rho_s\rho_n r e^{-2\rho_r\rho_s\rho_n r(n_G - n_G^*)}, \quad n_G^* \leq n_G < \infty. \quad [75]$$

which gives the temperature for the bacteria would be

$$\theta_B = \frac{1}{2\rho_r\rho_s\rho_n r}, \quad [76]$$

It is not surprise that factors such as the phage-bacteria HGT rate, phage population size, and burst size promote the "kill-the-winner" mechanism.

Then the critical "temperature" between Regime I/II is given by

$$N_B/\theta_B = 2r_V N_B \rho_s \rho_n \sim L. \quad [77]$$

However, this is not consistent with the collapse of the curves shown in Fig. 5. This perhaps results from two reasons: 1. the interference among booming strains in the boom-bust dynamics introduces complex self-consistency relations between total population size and other model parameters. 2. the extreme population fluctuations in boom-bust cycles also leads to inaccuracy in stochastic simulations. We would leave them as the future work. Nevertheless, our analytical approach provides qualitative insights into the system's behavior across a broader parameter space.

A.3. Summary. From the above calculations in Regime II&III, we can see that these ratios cannot be simply canceled out through parameter rescaling because they may affect demographic noises and HGT processes non-trivially. However, qualitatively, when ρ_r, ρ_s, and ρ_n are perhaps greater than 1, it implies the phage tends to kill the booming bacterium earlier, thereby reducing fluctuations in bacterial abundances and lowering the temperature. For instance, an increase in the phage's HGT rate or population size can enhance its establishment probability due to the larger rebirth rate. As a consequence, we can expect the setting of $\rho_r, \rho_s, \rho_n > 1$ to shift the transition to a lower value of the bacterial HGT rate r, compared to the baseline case where ρ_r, ρ_s, and ρ_n are set to 1, as shown in Fig. S8. In essence, the natural settings ($\rho_r, \rho_s, \rho_n > 1$) promote the gene and genotype diversity, compared to our simplified settings ($\rho_r = \rho_s = \rho_n = 1$).

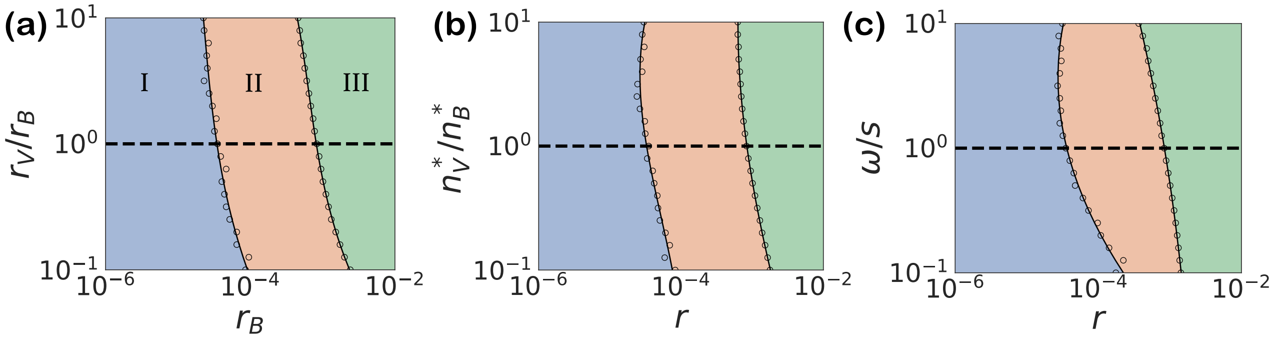

Fig. S8. Phase diagrams for phages and bacteria with different ratios of HGT rates, fixed points, and phage death rate/bacterial birth rate. The scatter points are the minimal HGT rates to keep gene and genotype diversity invariant over a simulation period $T = 250000$. The black solid lines are spline interpolation through the scatter points. The horizontal dashed line corresponds to the simplified case shown in Fig.4 in the main text.

In nature, the phage/host population ratio can be as much as 10. For biological plausibility, we further test our theory with the phage/bacteria population size ratio: $\rho_n = 10$, the burst size: $\rho_s * \rho_n$ =100 and the phage/bacteria HGT ratio $\rho_r = 0.1$ (we have to carefully choose alpha otherwise the "killing-the-winner" mechanism is so strong that the bacteria is hard to extinct). Fig. S9 shows that our theory actually gives an upper bound of the critical HGT rates which the model with realistic parameters would fall below.

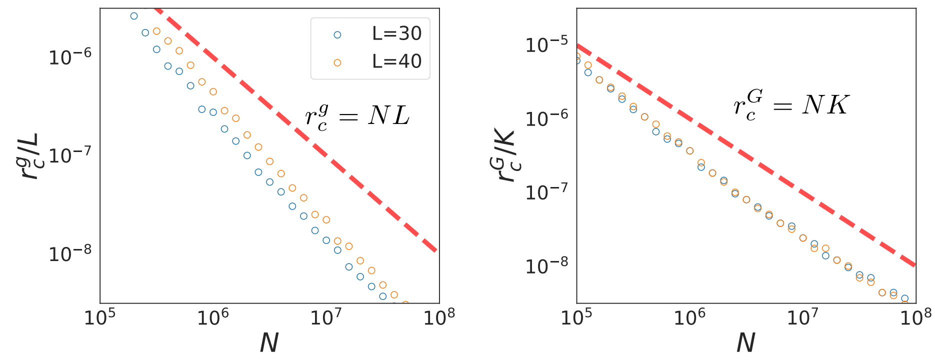

Fig. S9. Critical (minimal) HGT rates for gene and genotype coexistence for the non-symmetric parametrization. The simulation parameter set: the phage/bacteria population size ratio: $\rho_n = 10$, the burst size: $\beta = \rho_s * \rho_n$ =100 and the phage/bacteria HGT ratio $\rho_r = 0.1$.

B. Heterogeneous parameters. In the main text, we also assume that n_G and s are identical for all phage-bacteria pairs. We consider the heterogeneous case where s and n_G are sampled from the lognormal distribution for each phage-bacteria pair:

$$\rho(x) = \frac{1}{\sigma x\sqrt{2\pi}} e^{-\frac{(\log x)^2}{2\sigma^2}}. \quad [78]$$

Fig. S10 shows that the results derived from our simplified model with identical phage-bacteria pairs are robust to heterogeneity and work pretty well for $\sigma < 0.5$.

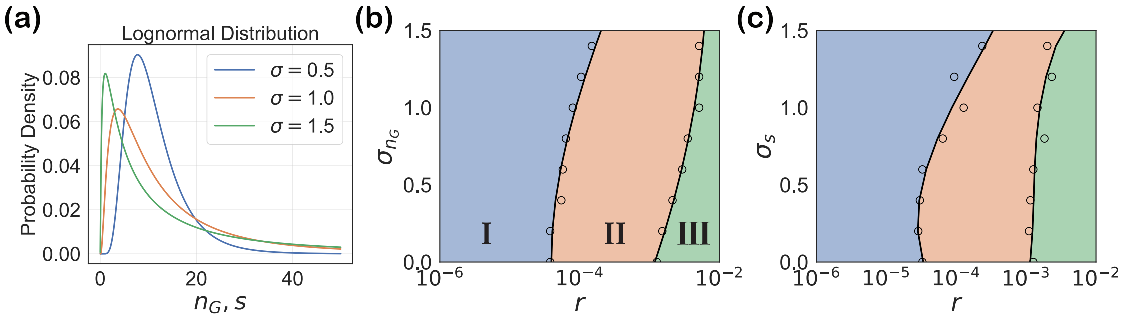

Fig. S10. Phase diagrams for s and n_G sampled from the log-normal distribution. The scatter points are the minimal HGT rates to keep gene and genotype diversity invariant over a simulation period $T = 250000$. The black solid lines are spline interpolation through the scatter points. $\sigma_n = 0$ and $\sigma_s = 0$ correspond to the simplified case shown in Fig.4 in the main text.

9. Beyond one-bacteria-one-phage infection

We extended our model to handle multiple phage-host infections by initializing a diagonal binary infection matrix first. We then generalized the interaction to allow a single phage strain to simultaneously infect multiple hosts, with the number of infections drawn from a Poisson distribution with a Poisson rate of 5, as illustrated in Fig. S11 Panel (a). Fig. S11 Panels (b) and (c) demonstrate that our scaling relations remain consistent. Furthermore, Fig. S11 Panel (d) illustrates the robustness of our results across a wide range of average infection numbers by systematically varying the Poisson rate.

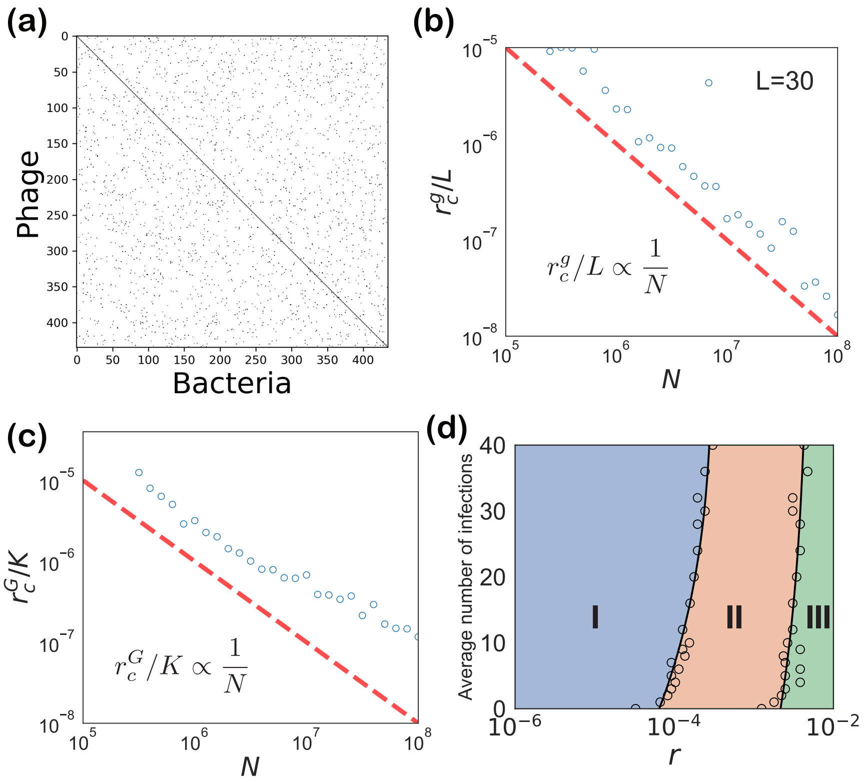

Fig. S11. **(a)** The heatmap for the binary sparse interaction matrix with $L = 40$. Each phage strain has the ability to infect another several random bacterial strains beside the exact matching one. The number of infections is sampled from the Poisson distribution with rate of 5. The y/x-axis are the labels of phages/bacteria. The yellow points are the non-zero entries, indicating the phage can infect that bacterium. **(b, c)** Minimal HGT rates for gene and genotype coexistence for inter-bacteria and phage-bacteria HGT. The red dashed line is our theoretical prediction, which shows our theory can also be generalized to the sparse interaction structure. **(d)** shows how the phase diagram is distorted by varying the average number of infections, i.e., the Poisson rate. Here we fix $N = 10^6$ and $L = 40$.

10. Transformation between the time and population distributions

In the bacterial booming process, the bacterial abundance grows exponentially with time:

$$n(t) = n_0 e^{st}, \quad dn = sndt, \tag{79}$$

where s is its fitness, and n_0 is the initial population size at $t = 0$.

We would like to estimate the population distribution from the time distribution,

$$Q(n) = \int P(n|t)P(t)dt = \int \delta(n_0 e^{st} - n)P(t)dt = \int \delta(t - \frac{1}{s}\log\frac{n}{n_0})P(t)\frac{dt}{sn} = \frac{1}{sn}P(\frac{1}{s}\log\frac{n}{n_0}). \tag{80}$$

Reverse this process, and recall the normalization: $\int P(t|n)dt = 1$,

$$P(t) = \int P(t|n)Q(n)dn = \int sn_0 e^{st}\delta(n_0 e^{st} - n)Q(n)dn = sn_0 e^{st}Q(n_0 e^{st}). \tag{81}$$

11. Establishment probability with a constant fitness for two different processes

A. Birth-death process. When the phage population is small, its targeted bacterium's fitness is s. In a unit of time Δt, the bacterium's birth probability is $(1+s)\Delta t$, its death probability Δt, and the probability that neither happens is $1 - (s+2)\Delta t$. With the basic extinction theory of branching processes, its extinction probability after t generations, $w(t)$, follows

$$w(t) = (1 - (s+2)\Delta t)w(t-1) + \Delta t + (1+s)\Delta t w(t-1)^2, \tag{82}$$

where we assume each birth event happens with two offspring. In the continuum limit,

$$\frac{dw}{dt} = 1 - (s+2)w + (1+s)w^2. \tag{83}$$

It is useful to rewrite in terms of the survival probability $\phi(t) = 1 - w(t)$:

$$\frac{d\phi}{dt} = s\phi - (1+s)\phi^2. \tag{84}$$

In the long-time limit, it gives the establishment probability

$$\phi(\infty) = \frac{s}{1+s} \approx s. \tag{85}$$

B. Poisson process. In our simulation, we use the Poisson process instead of the birth-death process with two offspring. Instead of eq. (82), the extinction probability after time t follows

$$w(t) = e^{-(s+1)\Delta t} \sum_{k=0}^{\infty} \frac{((s+1)\Delta t)^k}{k!} w(t-\Delta t)^k = e^{(s+1)\Delta t[w(t-\Delta t)-1]}, \tag{86}$$

where $e^{-(s+1)\Delta t} \frac{(s\Delta t)^k}{k!}$ is the probability of having k offspring during a unit of time Δt, and $w(t-\Delta t)^k$ is the probability that all k offspring go extinct after $t-\Delta t$.

The expression in terms of the survival probability is

$$1 - \phi(t) = e^{-(1+s)\Delta t \phi(t-\Delta t)}. \tag{87}$$

Assuming $\phi(\infty)$ is small, and choosing $\Delta t = 1$ in the simulation, the establishment probability is

$$\phi(\infty) = \frac{2s}{1+2s} \approx 2s. \tag{88}$$

Comparing with eq. (85), our simulation set-up brings a factor of 2. We use the birth-death process in the weak HGT regime because it is easy to construct the partial differential equation (PDE) in the continuum limit. When comparing with the simulation, we will correct our theoretical results about the establishment probability by a factor of 2.

12. Other supplementary figures

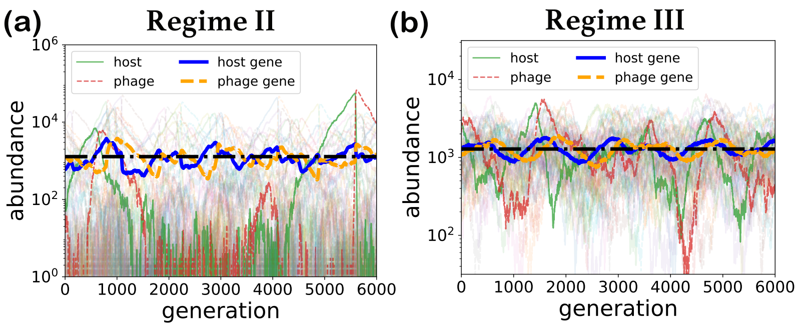

Fig. S12. Comparison between gene and genotype dynamics in Regime II (**a**) and Regime III (**b**). We show the population dynamics of one specific gene and multiple genotypes containing that gene. In order to compare gene and genotype abundance at the same scale, the gene abundance is divided by $L-1$. The black dash-dotted line is the average of genotype abundances.

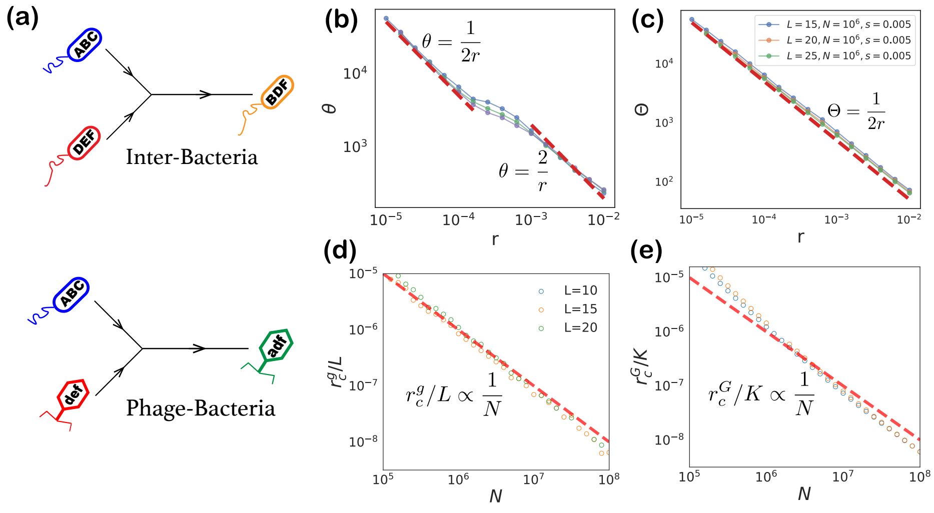

Fig. S13. $(\mathbf{a})$ Scheme of inter-bacteria and phage-bacteria HGT for "tripleton". The phage and bacterium can carry three different genes. There is one gene transferred in each HGT event. $(\mathbf{b})$ Effective genotype temperature Θ and $(\mathbf{c})$ gene temperature θ for "tripleton". Critical HGT rates for $(\mathbf{d})$ gene coexistence and $(\mathbf{e})$ genotype coexistence for the "tripleton" case. The red dashed lines are our theoretical predictions (the same as "doubleton" case).

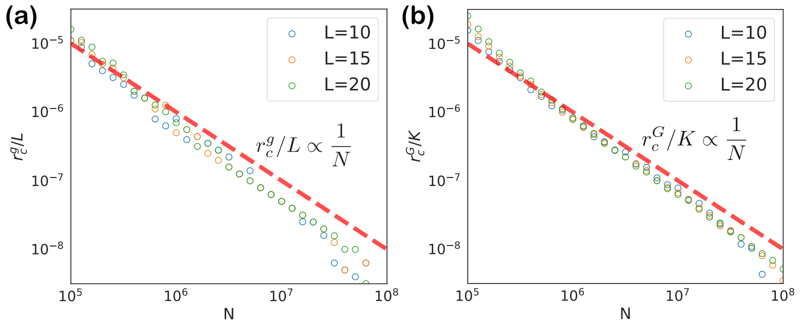

Fig. S14. Minimal HGT rates for gene coexistence $(\mathbf{a})$ and genotype coexistence $(\mathbf{b})$ for inter-bacteria and phage-bacteria HGT without fixing the total population size. The red dashed line is our theoretical prediction, which shows the constraint of fixing the total population size does not affect our results in the main text.

References

1. DT Gillespie, Approximate accelerated stochastic simulation of chemically reacting systems. *The J. chemical physics* **115**, 1716–1733 (2001).
2. RA Neher, BI Shraiman, DS Fisher, Rate of adaptation in large sexual populations. *Genetics* **184**, 467–481 (2010).
3. EH Kerner, A statistical mechanics of interacting biological species. *The bulletin mathematical biophysics* **19**, 121–146 (1957).
4. NS Goel, SC Maitra, EW Montroll, On the volterra and other nonlinear models of interacting populations. *Rev. modern physics* **43**, 231 (1971).
5. A Dobrinevski, E Frey, Extinction in neutrally stable stochastic lotka-volterra models. *Phys. Rev. E* **85**, 051903 (2012).
6. CW Gardiner, , et al., *Handbook of stochastic methods.* (springer Berlin) Vol. 3, (1985).
7. MT Pearce, A Agarwala, DS Fisher, Stabilization of extensive fine-scale diversity by ecologically driven spatiotemporal chaos. *Proc. Natl. Acad. Sci.* **117**, 14572–14583 (2020).
8. B Ottino-Löffler, M Kardar, Population extinction on a random fitness seascape. *Phys. Rev. E* **102**, 052106 (2020).
9. T Agranov, G Bunin, Extinctions of coupled populations, and rare event dynamics under non-gaussian noise. *Phys. Rev. E* **104**, 024106 (2021).
10. RA Neher, CA Russell, BI Shraiman, Predicting evolution from the shape of genealogical trees. *Elife* **3**, e03568 (2014).
11. MM Desai, DS Fisher, Beneficial mutation–selection balance and the effect of linkage on positive selection. *Genetics* **176**, 1759–1798 (2007).
12. DL Snyder, MI Miller, *Random point processes in time and space.* (Springer Science), (2012).
13. RA Neher, BI Shraiman, Statistical genetics and evolution of quantitative traits. *Rev. Mod. Phys.* **83**, 1283 (2011).
